# The antiviral drug telaprevir induces cell death by reducing *FOXA1* expression in estrogen receptor α (ERα)‐positive breast cancer cells

**DOI:** 10.1002/1878-0261.13303

**Published:** 2022-09-03

**Authors:** Stefania Bartoloni, Stefano Leone, Sara Pescatori, Manuela Cipolletti, Filippo Acconcia

**Affiliations:** ^1^ Department of Sciences, Section Biomedical Sciences and Technology University Roma TRE Italy

**Keywords:** breast cancer, estrogen receptor α, FOXA1, telaprevir

## Abstract

Previously, we found that telaprevir (Tel), the inhibitor of hepatitis C virus NS3/4A serine protease, reduces estrogen receptor α (ERα) content at the transcriptional level without binding to the receptor, prevents ERα transcriptional activity, and inhibits basal and 17β‐estradiol (E2)‐dependent cell proliferation in different breast cancer (BC) cell lines. Here, we further characterize the Tel action mechanisms on ERα levels and function, identify a possible molecular target of Tel in BC cells, and evaluate Tel as an antiproliferative agent for BC treatment. Tel‐dependent reduction in ERα levels and function depends on a Tel‐dependent decrease in FOXA1 levels and activity. The effect of Tel is transduced by the IGF1‐R/AKT/FOXA1 pathway, with the antiviral compound interacting with IGF1‐R. Tel prevents the proliferation of several BC cell lines, while it does not affect the proliferation of normal nontransformed cell lines, and its antiproliferative effect is correlated with the ratio of FOXA1/IGF1‐R expression. In conclusion, Tel interferes with the IGF1‐R/AKT/FOXA1 pathway and induces cell death in ERα‐expressing BC cells. Thus, we propose that this antiviral could be repurposed for the treatment of ERα‐expressing BC.

## Graphical abstract

Telaprevir prevents the growth of breast cancer cell lines by interfering with the IGF‐1R pathway leading to FOXA1 down‐regulation, ERα inhibition, and induction of apoptotic cell death.

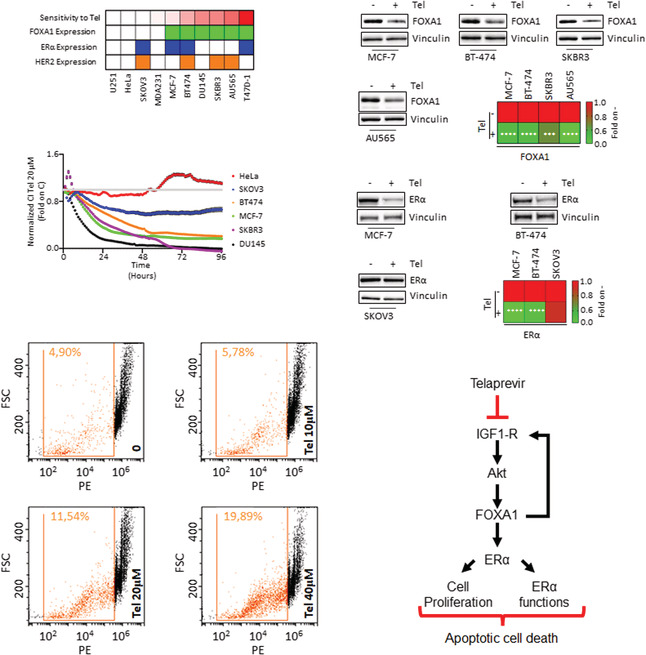

**Abbreviations**
Abe: abemaciclib
AKT: v‐akt murine thymoma viral oncogene homolog 1 (AKT)
BC: breast cancer
CDK4: cyclin‐dependent kinase 4
CDK6: cyclin‐dependent kinase 6
CI: combination index
CREB: cyclic AMP‐responsive element‐binding protein 1
DAPI: 4′,6‐diamidino‐2‐phenylindole
DARTS: drug affinity responsive target stability
DMEM: Dulbecco's midified modified eagle medium
DMSO: dimethyl sulfoxide
E2: 17β‐estradiol
EGF: epidermal ggrowth factor
ERBB2: V‐Erb‐B2 avian erythroblastic leukemia viral oncogene homolog 2
ERE: estrogen responsive element
ERK: extracellular regulated kinases
ERα: estrogen receptor α
ET: endocrine therapy
FDA: food and drugs administration
FOXA1: forkhead box A1
HCV: hepatitis C virus
HER2: human epidermal growth factor receptor 2
IC_50_
: inhibitor concentration 50
IGF: insulin‐like growth factor
IGF1‐R: insulin‐like growth factor 1 receptor
MBC: metastatic breast cancer
Palbo: palbociclib
PARP: poly (ADP‐ribose) polymerase
PBS: phosphate‐buffered saline
PI3K: phosphatidyl‐inositol‐3‐kinase
pS2: presenelin2
STS: staurosporine
Tel: telaprevir
TFF1: trefoil factor 1
YY buffer: Yos Yarden

## 1 Introduction

The 11.7% of all cancer cases are represented by female breast cancer (BC) [1], which is the most common cancer in women worldwide [2]. Breast cancer is a heterogeneous disease characterized by different molecular markers and clinical features, but, approximatively, 75% of BC cases are 17β‐estradiol (E2)‐dependent and express the estrogen receptor α (ERα), which sustains tumor resilience and growth [3]. Therefore, ERα protein is a leading therapeutic target and, thereby, the mainstream treatment for all ERα‐expressing BCs is endocrine therapy (ET). Endocrine therapy includes selective estrogen receptor modulators (e.g., tamoxifen) and selective estrogen receptor downregulators (e.g., fulvestrant), which alter ERα levels and perturbs receptor signaling, as well as aromatase inhibitors, which indirectly block ERα by estrogen deprivation [2]. However, ET effectiveness is limited by the development of endocrine resistance, which in turn leads to disease recurrence or progression to a metastatic setting [4]. Therefore, a novel therapeutic strategy that overcomes ET limitation is needed.

Previously, we proposed the modulation of ERα intracellular levels, more than ERα protein itself, as a druggable target [5]. Indeed, several lines of evidence indicate that factors perturbing the mechanisms involved in the modulation of ERα content could prevent ERα‐mediated proliferation of BC cells. These factors not only include synthetic ERα ligand (e.g., fulvestrant and tamoxifen) [6, 7], but also molecules that do not bind to ERα (e.g., emetine, carfilzomib, ouabain, and digoxin) [8, 9, 10, 11] and pathways not directly related to ERα signaling (e.g., endocytic and metabolic pathways) [12, 13, 14, 15, 16, 17], which in turn alter ERα levels and inhibit BC cells proliferation.

Therefore, the modulation of ERα intracellular levels can be used as a bait for the identification of novel molecules that directly or indirectly modulate ERα levels and functions, thus preventing BC progression [5]. Based on this principle, we performed a screening of food and drugs administration (FDA)‐approved drugs in MCF‐7 cells and found telaprevir (Tel) to reduce ERα levels and to inhibit MCF‐7 cell proliferation [10].

Tel is an antiviral agent that inhibits hepatitis C virus (HCV) NS3/4A serine protease [18] and, to date, no data regarding Tel effect on BC exist but we recently demonstrated that Tel reduces ERα levels by affecting receptor expression at transcriptional levels, perturbs E2:ERα signaling by inhibiting receptor transcriptional activity, and prevents basal and E2‐dependent cell proliferation in different BC cell lines [19]. However, at the present, the molecular mechanism(s) through which Tel acts as a selective modulator of ERα levels and degradation and as an antiproliferative agent for BC cells is not known.

Here, we dissected the molecular mechanism(s) of Tel action on both ERα levels and functions and cell proliferation to identify Tel molecular target(s) in BC cells. The results reported here indicate that Tel interferes with the IGF1‐R/AKT/FOXA1 pathway and induces cell death in ERα‐expressing BC cells.

## 2 Materials and methods

### 2.1 Cell culture and reagents

Our laboratory acquired the following reagents (i.e., Dulbecco's modified Eagle's medium (DMEM, with and without phenol red), McCoy's 5a medium modified, RPMI‐1640 medium, NVP AEW541 (NVP), DAPI (D‐9542), staurosporine (STS), and fetal calf serum) from Sigma‐Aldrich (St. Louis, MO, USA) and the Bradford protein assay kit, anti‐mouse, and anti‐rabbit secondary antibodies from Bio‐Rad (Hercules, CA, USA). Human fibroblasts were a generous gift of Prof. Valentina Pallottini, University Roma TRE, Rome, Italy. MCF10a, MDA‐MB‐231, MCF‐7, T47D‐1, BT‐474, SKBR3, AU565, HeLa, SKOV3, and DU145 cells were purchased by ATCC (Manassas, VA, USA) and maintained according to the manufacturer's instructions. U251 cells were a generous gift of Dr Francesco Berardinelli, University Roma TRE, Rome, Italy. Antibodies against ERα (HC‐20, rabbit), AKT1 (B‐1, mouse), and pS2 (FL‐84, rabbit) were obtained from Santa Cruz Biotechnology (Santa Cruz, CA, USA). Anti‐vinculin, anti‐tubulin, and anti‐FLAG antibodies were purchased from Sigma‐Aldrich. Antibodies against FOXA1/HNF3α (53528S, rabbit), phospho‐AKT Ser473 (4058S, rabbit), PARP (9542T, rabbit), phospho‐IGF1‐R (3024S, rabbit), and IGF1‐R (3027S, rabbit) were purchased by Cell Signaling Technology (Danvers, MA, USA). Chemiluminescence reagent for western blotting was obtained from Bio‐Rad Laboratories. Fulvestrant (i.e., faslodex or ICI 182780) and 4OH‐tamoxifen (Tam) were purchased from Tocris (Bristol, UK). Telaprevir (VX‐950, Tel), palbociclib (Palbo), and abemaciclib (Abe) were purchased from Selleck Chemicals (Houston, TX, USA). Triciribine, MK‐2206, AG‐879, and AG‐825 were purchased from Cayman Chemical Company (Ann Arbor, MI, USA). PD‐98059 (PD), KG‐501 (KG), and PPP were obtained from Calbiochem. Promega (Madison, MA, USA) supplied the Nano‐Glo^®^ EndurazineTM. All the other products were obtained from Sigma‐Aldrich. Analytical‐ or reagent‐grade products were used without further purification.

### 2.2 Cellular and biochemical assays

Cells were grown in 10% serum and either DMEM with phenol red, McCoy's 5a modified or RPMI‐1640 medium for 24 h and then treated with either telaprevir (Tel), NVP, MK‐2206 at the indicated doses and for the indicated periods. For the IGF and EGF experiments, cells were grown in DMEM with phenol red plus 1% charcoal‐stripped fetal calf serum for 24 h before Tel, NVP, gefitinib (Gef), IGF, and EGF administration at the indicated doses and for the indicated time points. Cell lysis western blotting analyses were performed as previously reported in [9, 20, 21, 22].

### 2.3 In‐cell western blotting

Five thousand MCF‐7 cells were plated in a 96‐well plate in a sextuplicate for each treatment condition. The next day, cells were treated with vehicle (DMSO) or 1 μm of NVP, triciribine, AG‐879, PD‐98059, and KG‐501 for 48 h. siRNA‐mediated FOXA1 depletion was used as the internal control. The procedure was followed as previously reported in [9, 20, 21, 22, 23] except that the antibody against FOXA1 was used 1 : 5000 in BSA2%/PBS.

### 2.4 Growth curves

Growth curves and drug synergy studies were done as previously reported in [9, 19, 21, 22, 24], and the synergy was calculated with combenefit freeware software [25] while the combination index (CI) was calculated as previously reported [26, 27].

### 2.5 Apoptosis analysis

For hypodiploid peak analysis, Nicoletti's protocol was followed [28]. Briefly, cell pellet was resuspended in 500 μL of PBS + 500 μL of hypotonic staining solution (0.1% sodium citrate (w/v), 0.1% Triton X‐100 (v/v), 50 μg·mL^−1^ propidium iodide, pH 7.8). Cells were incubated for 30 min at room temperature. Finally, 20 000 total events, presented in a logarithmic scale (FL‐2 Vs FSC) were acquired directly in the staining buffer, and percentage of the hypodiploid peak was calculated by a proper electronic marker. Measurement of caspase 9 activity was performed by employing the Caspase‐Glo^®^ 9 assay system (Promega) according to the manufacturer's instructions. Activity was acquired at a Tecan Spark microplate reader (Männedorf, Switzerland). Light emission was continuously measured for 2 h every 5 min; ratio between the final (specific) and the initial (nonspecific) values was used to quantitate the effects of the tested drugs.

### 2.6 Real‐Time measurement of ERα and FOXA1 transcriptional activity

For the analyses of ERα transcriptional activity, MCF‐7 cells stably expressing an ERE‐nanoluciferase (NLuc)‐PEST reporter gene were used as previously reported [21, 22]. For the analyses of FOXA1 activity on its specific enhancer in the promoter of ESR1 gene, MCF‐7 cells were stably transfected with pGL2Basic Neo_NLucP_ESR1_(FOXA1) plasmid [29] using Lipofectamine 2000 (Thermo Fisher Scientific, Waltham, MA, USA) reagent according to the manufacturer's instructions. The transfection medium was changed after 24 h, and G418 (500 μg·mL^−1^) was added for the selection of MCF‐7 ESR1‐NLuc cells. Pooled clones were used for the experiments. The selection antibiotic was left in the growing medium while each experiment was performed in the absence of antibiotics. MCF‐7 ESR1‐NLuc cells were seeded in 96‐well plate (5000 cells per well) and 24 h after plating cells were treated with Tel, NVP, triciribine, and PPP at the indicated doses and for the indicated time points. For MCF‐7 ESR1‐NLuc cells depleted of FOXA1 expression by siRNA, 3 h after the second transfection cells were detached, seeded in 96‐well plate (5000 cells per well), and treated with Tel for the indicated doses and time points. After treatment, Nano‐Glo^®^ EndurazineTM was added according to the manufacturer's instruction in 50 μL as the final experimental volume. Plates were then transferred into a Tecan Spark microplate reader. Light emission (released light units [RLU]) was measured for 24 h every 5 min.

### 2.7 Small interference RNA (siRNA) experiments

Silencing of FOXA1 in MCF‐7, MCF‐7 ESR1‐NLuc, and SKBR3 cells and silencing of ERα in MCF‐7 and SKOV3 cells were achieved via transient transfection using Dharmacon Smartpool siRNA ERa siRNA (final concentration 4 nm in 2 mL of a six‐well plate), with Lipofectamine RNAiMAX (Invitrogen, Waltham, MA, USA; 6 μL per well of a six‐well plate), following the manufacturer's instructions. Cells were subjected to double transfection: ‘reverse’ (cells in suspension) on day 1 and ‘forward’ (adherent cells) on day 2. The medium was changed after 3 h from both transfections. Cells were then processed and analyzed either 24, 48, or 72 h after the second transfection.

### 2.8 Confocal microscopy analysis

MCF‐7 cells were seeded (10 000 cells per well) in 8‐well chamber slide. Twenty‐four hours after plating, cells were treated with Tel (from 10 to 40 μm) for 48 h and then were fixed in 70% ETOH. After washing in phosphate‐buffered saline (PBS), cells were stained with DAPI (Sigma‐Aldrich) in PBS at a final concentration of 2 μg·mL^−1^ for 30 min at room temperature in the dark. Images were acquired by confocal microscopy using a 20× dry objective lens and a 63× oil immersion objective lens at 405 nm laser UV. Confocal analysis was performed using LCS (Leica Microsystems, Wetzlar, Germany).

### 2.9 Drug affinity responsive target stability (DARTS)

DARTS analysis was performed according to Lomenick's protocol [30, 31]. MCF‐7 cells were grown to approximately 80–85% confluence in a 10 cm dish and then lysed in YY buffer. The protein concentration of the lysate was measured with the Bradford method. The cell lysate was divided into identical aliquots and incubated with either vehicle (DMSO), Tel or NVP at the indicated doses for 1 h at room temperature. The aliquots of lysate were then treated with vehicle (TNC 10× buffer) or pronase (1 : 1000, Roche Applied Science, Indianapolis, IN, USA) for 30 min at room temperature. Subsequently, the proteolysis was stopped by adding a 4× loading solution and heating to 100 °C for 5 min. Next, the samples were subjected to western blotting analyses.

### 2.10 Statistical analysis

Statistical analysis and band quantitations were performed as previously reported [9, 20, 21, 22, 23]. Significant differences are calculated by the Student *t*‐test or with one‐way ANOVA followed by the Tukey post‐test using the instat version 8 software system (Graph‐Pad Software Inc., San Diego, CA, USA), and the *P* values are given in figure captions.

## 3 Results

### 3.1 Effect of telaprevir on the proliferation of nontransformed and transformed cell lines

**Fig. 1:**
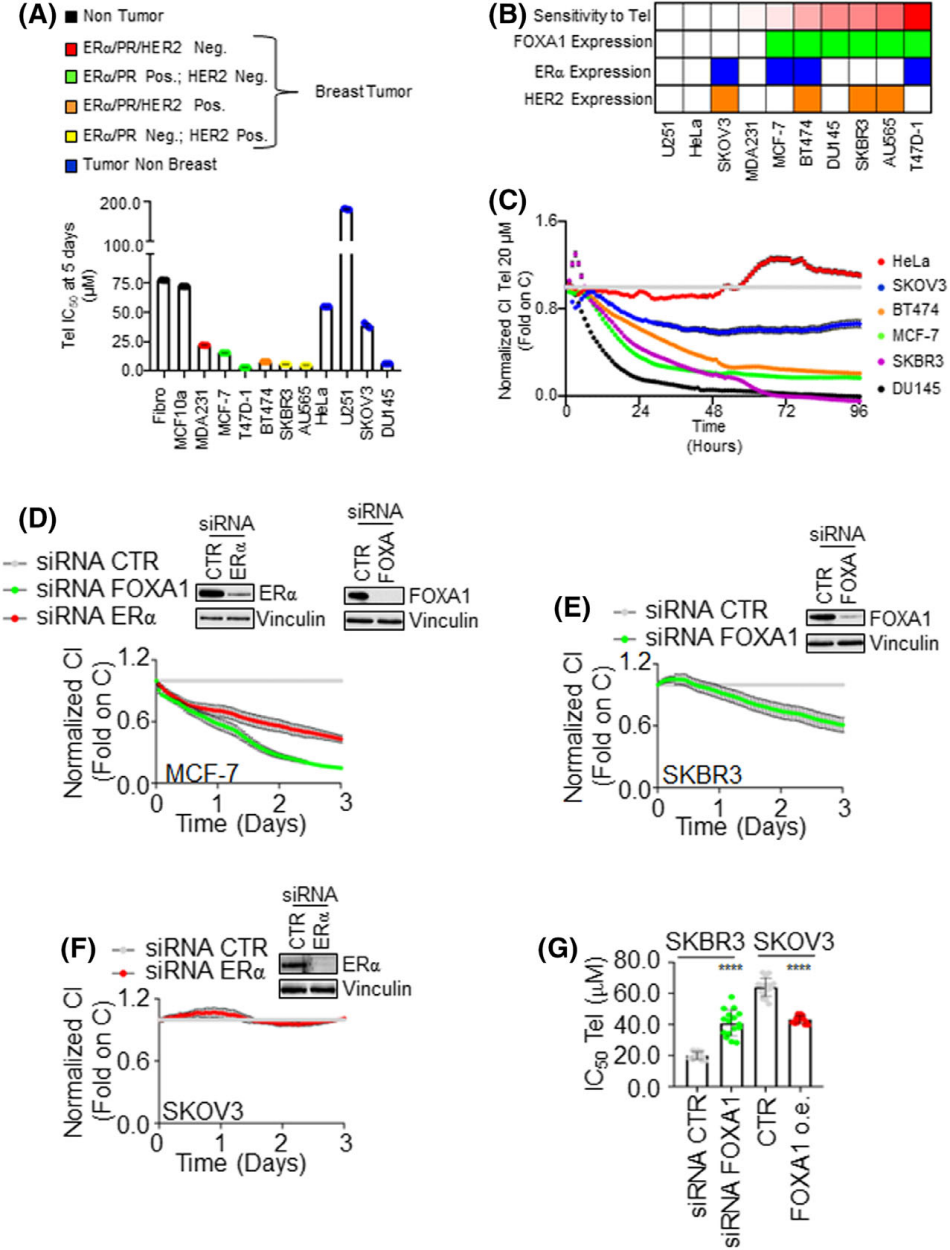
Effect of telaprevir on the proliferation of nontransformed and transformed cell lines. (A) Telaprevir (Tel) inhibitor concentration 50 (IC50‐μm) measured on a panel of nontransformed (i.e., human fibroblast and MCF10a cells) and transformed (i.e., MDA‐MB‐231, MCF‐7, T47D‐1, BT‐474, SKBR3, AU565, HeLa, U251, SKOV3, and DU145 cells) cell lines at 5 days. *N* = 4. (B) The panel indicates cell sensitivity to Tel antiproliferative effect (red) and ERα (blue), HER2 (orange), and FOXA1 (green) mRNA expression in the indicated cell lines. ERα, Her2, and FOXA1 expression was extrapolated *in silico* from the Depmap portal database (https://depmap.org/portal/). (C) Real‐time growth curves in the indicated cell lines treated with Tel 20 μm (0–96 h). Normalized cell index (i.e., CI) has been calculated at each time point with respect to the control sample (gray line). Data are the mean ± standard errors *n* = 8. Real‐time growth curve analyses in MCF‐7 (D), SKBR3 (E), and SKOV3 (F) cells depleted of ERα (red line) and/or FOXA1 (green line) by siRNA. Normalized cell index (i.e., CI) has been calculated at each time point with respect to the control sample (gray line). Data are the mean ± standard errors *n* = 8. (G) Tel inhibition concentration 50 (IC_50_‐μm) on SKBR3 and SKOV3 cells proliferation measured in the presence or the absence of FOXA1 expression at 3 days in SKBR3 cells and 5 days in SKOV3 cells. Data are the mean ± standard deviations with a *P*‐value < 0.0001. **** indicates significant differences with respect to the control sample (siRNA CTR or CTR) calculated with Student *t*‐test. *n* = 17. [Colour figure can be viewed at wileyonlinelibrary.com]

Previously, we found that telaprevir (Tel) reduces both the basal and the 17β‐estradiol (E2)‐induced proliferation in different ERα‐expressing breast cancer (BC) cell lines [19]. Thus, we next evaluated the Tel antitumor activity in different types of transformed and nontransformed cell lines. To this purpose, real‐time growth curve analyses were performed on a panel of nontransformed (i.e., human fibroblasts and MCF10a cells), breast tumor (i.e., MDA‐MB‐231, MCF‐7, T47D‐1, BT‐474, SKBR3, and AU565 cells), and non‐breast tumor (i.e., HeLa, U251, SKOV3, and DU145 cells) cell lines (Fig. 1A). Cells were treated with increasing doses (from nm to μm) of the antiviral, and the Tel inhibitor concentration 50 (IC_50_) was calculated at 5 days. The cell lines were considered sensitive to Tel antiproliferative effect when the calculated IC_50_ was lower than 20 μm, while the cell lines were considered resistant to the Tel effect if the calculated IC_50_ was higher than 20 μm. As shown in Fig. 1A, Tel antiproliferative effect is cell line‐dependent and selective for transformed cell lines, while Tel does not affect the proliferation of nontransformed cell lines. Notably, BC cell lines appear to be more sensitive to Tel antiproliferative effect rather than non‐breast tumor cell lines, except for the prostate cancer DU145 cells (Fig. 1A).

Next, the putative factors defining cell sensitivity to Tel antiproliferative effect were searched and ERα, human epidermal growth factor receptor 2 (HER2), and FOXA1 were selected as potential candidates since these proteins play a significant role in BC development and progression [32, 33, 34]. Thus, the mRNA expression levels of these proteins were compared *in silico* using the data available in the Cancer Dependency Map Project at Broad Institute database (Depmap portal at https://depmap.org/portal/interactive/) in different tumor cell lines (i.e., U251, HeLa, SKOV3, MDA‐MB‐231, MCF‐7, BT‐474, DU145, SKBR3, AU565, and T47D‐1 cells). Interestingly, the cell sensitivity to Tel antiproliferative effect does not correlate with ERα or HER2 mRNA expression, while correspondence between Tel sensitivity and the expression of FOXA1 mRNA was observed (Fig. 1B). To validate this observation, real‐time growth curve analyses were performed in some of the tested cell lines. The results indicate that Tel significantly affects the proliferation of the cell lines expressing FOXA1 mRNA (i.e., BT‐474, MCF‐7, SKBR3, and DU145 cells), but the antiviral does not affect the proliferation of those cell lines devoid of FOXA1 (i.e., HeLa and SKOV3 cells; Fig. 1C). These observations suggest that Tel antiproliferative activity could be correlated with FOXA1 expression in cancer cells.

Consequently, the differential impact of ERα and FOXA1 on cell proliferation was next evaluated. For this purpose, three different cellular models were selected: MCF‐7, SKOV3, and SKBR3 cells that, respectively, express both FOXA1 and ERα, only ERα, and only FOXA1 (Fig. S1A). The effect of siRNA‐dependent ERα and/or FOXA1 depletion on cell proliferation was analyzed in these model systems. The results indicate that FOXA1 depletion reduced MCF‐7 and SKBR3 cell proliferation (Fig. 1D,E). Notably, reduction in FOXA1 levels affects MCF‐7 cell proliferation more than the siRNA‐mediated ERα intracellular level reduction (Fig. 1D). In contrast, ERα depletion does not impact on SKOV3 cell proliferation (Fig. 1F). These data suggest that FOXA1 is upstream to ERα in the control of cell proliferation.

Next, to understand the FOXA1 involvement in Tel antiproliferative effect, the expression of FOXA1 was reduced in SKBR3 cells, while SKOV3 cells stably overexpressing FOXA1 were produced (Fig. S1B), different doses of Tel were administered in these cellular models, and cell proliferation was measured. The Tel IC_50_ was significantly increased in SKBR3 cells where FOXA1 expression was reduced with respect to control SKBR3 cells, while Tel IC_50_ was significantly reduced in SKOV3 cells overexpressing FOXA1 with respect to the parental cell line (Fig. 1G and Fig. S1C,D).

Overall, these results imply that FOXA1 expression is required, at least in part, for the Tel antiproliferative effect.

### 3.2 FOXA1 involvement in telaprevir‐dependent regulation of ERα levels and functions

**Fig. 2:**
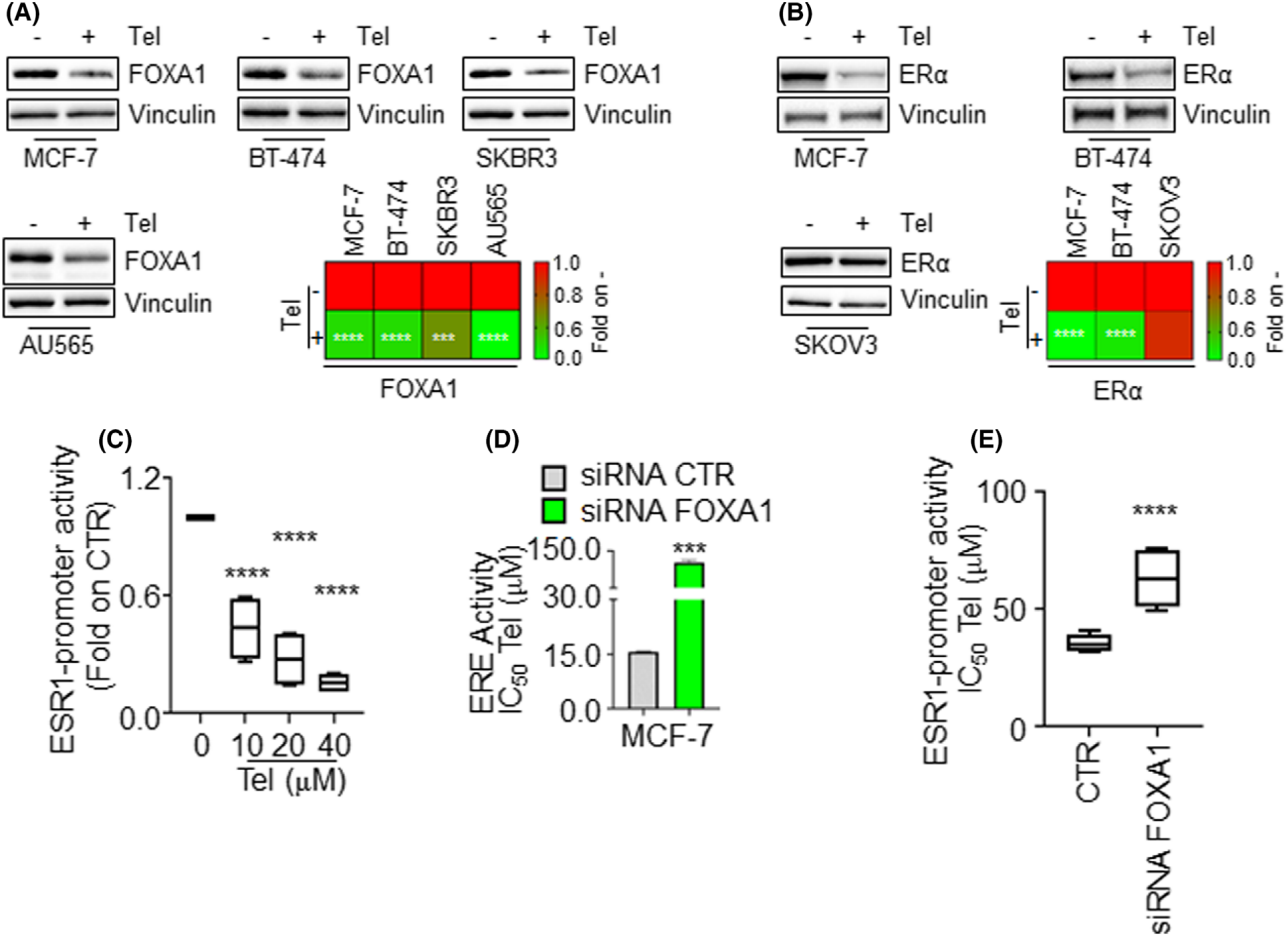
FOXA1 involvement in telaprevir effect on ERα levels and functions. (A) Western blotting and relative quantitation (heat map) of FOXA1 levels in MCF‐7, BT‐474, SKBR3, and AU565 cells treated with telaprevir (Tel 20 μm) for 72 h. The loading control was done by evaluating vinculin expression in the same filter. (B) Western blotting and relative quantitation (heat map) of ERα and vinculin levels in MCF‐7, BT‐474, and SKOV3 cells treated with Tel 20 μm for 72 h. Panels show representative blots. Histograms are reported in Fig. S3A,B, where the mean ± standard deviation with a *P*‐value < 0.001 (***) and 0.0001 (****) is reported. * indicates significant differences with respect to the control sample (−) of each cell line and has been calculated with Student *t*‐test. *n* = 4 for all the experiments while *n* = 6 for SKBR3 cell lines. The activity of FOXA1‐specific enhancer in ESR1 promoter region measured in MCF‐7 ESR1‐NLuc cells treated with the indicated concentrations of Tel for 48 h (C) or with the FOXA1 siRNA (E). Data are the mean ± standard deviation with a *P*‐value < 0.0001 (****). * indicates significant differences with respect to the control sample (0) calculated with one‐way ANOVA followed by the Tukey post‐test (panel C) and with Student *t*‐test (panel E), *n* = 10. (D) ERE promoter activity in MCF‐7 ERE‐NLuc cells treated with the indicated doses of Tel for 48 h both in the absence or presence of FOXA1 siRNA. *n* = 10. Data are the mean ± standard deviation with a *P*‐value < 0.001 (***). * indicates significant differences with respect to the control sample (CTR) calculated with Student *t*‐test. [Colour figure can be viewed at wileyonlinelibrary.com]

Because FOXA1 is required for ERα expression in BC cells [35] and Tel reduces ERα levels in different BC cell lines [19], FOXA1 siRNA‐mediated depletion on ERα levels phenocopies the effect of Tel on receptor levels (Fig. S2A). Therefore, because Tel reduces FOXA1 mRNA levels (data not shown), we tested the possibility that a Tel‐dependent reduction in FOXA1 levels could be required for Tel‐dependent reduction of ERα levels. For this purpose, the Tel effect on FOXA1 and ERα protein levels was analyzed in different cell lines expressing both proteins (MCF‐7 and BT‐474 cells), only FOXA1 (SKBR3 and AU565 cells) and only ERα (SKOV3 cells). Results show that Tel (20 μm) reduced FOXA1 levels in MCF‐7, BT‐474, SKBR3, and AU565 cells (Fig. 2A) and ERα levels in MCF‐7 and BT‐474 cells within 72 h (Fig. 2B and Fig. S3A). Notably, Tel does not affect ERα levels in SKOV3 cells (Fig. 2B and Fig. S3B), which do not express FOXA1. Therefore, present results suggest that FOXA1 could be required for Tel‐dependent reduction in ERα intracellular content.

To further demonstrate that the Tel‐dependent reduction in receptor intracellular levels was due to the ability of the antiviral to reduce FOXA1 expression, we generated an MCF‐7 cell line stably expressing the FOXA1‐specific enhancer of the ESR1 promoter region (i.e., the genetic region in the ERα gene promoter required for FOXA1‐dependent ERα expression) [29] upstream to a nanoluciferase (NLuc)‐PEST reporter gene (MCF‐7 ESR1‐NLuc cells). In these cells, Tel reduced ESR1 promoter activity (i.e., FOXA1 activity) in a dose‐dependent manner (Fig. 2C). Altogether these findings demonstrate that Tel‐dependent ERα reduction directly relies on Tel‐dependent decrease in FOXA1 levels and function.

Because Tel prevents FOXA1 functions and affects ERα transcriptional activity [19], and FOXA1 is a pioneer factor required for ERα transcriptional activity [36], we next evaluated the FOXA1 requirement in Tel ability to interfere with ERα transcriptional activity. For this purpose, we used the MCF‐7 ERE‐NLuc cells, where a 3xERE‐TATA reporter construct was stably introduced upstream of the NLuc‐PEST reporter gene [21, 22]. As expected, reduction in FOXA1 levels strongly reduced basal ERE promoter activity (Fig. S2B), as well as ERα expression and basal expression of presenilin 2 (pS2), an ERE‐containing ERα target gene, in MCF‐7 cells (Fig. S2C). Next, MCF‐7 ERE‐NLuc cells were challenged for 48 h with different doses of Tel both in the presence and the absence of the FOXA1 siRNA‐mediated depletion, and the Tel IC_50_ for ERα transcriptional activity was measured. Tel‐dependent reduction in ERα transcriptional activity is diminished when FOXA1 expression is down‐modulated in MCF‐7 ERE‐NLuc cells (Fig. 2D). Accordingly, the ability of Tel to reduce pS2, ERα, and FOXA1 levels in parental MCF‐7 cells was impaired when FOXA1 was depleted (Fig. S2D,D’,D”,D”’). These data indicate that Tel‐dependent reduction in FOXA1 levels controls Tel‐induced decrease in ERα transcriptional activity.

To further demonstrate that FOXA1 transcriptional activity is specifically involved in Tel‐dependent reduction in ERα expression, MCF‐7 ESR1‐NLuc cells were challenged for 48 h with different doses of Tel both in the presence and the absence of the FOXA1 siRNA‐mediated depletion (Fig. S2E) and the Tel IC_50_ for FOXA1 transcriptional activity was measured. Depletion of FOXA1 impaired the Tel ability to diminish in a dose‐dependent manner the FOXA1 transcriptional activity on ESR1 promoter enhancer (Fig. 2E).

Altogether these data indicate that Tel impact on FOXA1 levels and activity is upstream of the effect of Tel on ERα expression and transcriptional function.

### 3.3 Action mechanism for the telaprevir‐induced reduction in FOXA1 levels

At present, there are no data regarding the signal transduction pathways involved in Tel‐mediated regulation of FOXA1 levels and function in BC cells. However, a role for insulin‐like growth factor 1 receptor (IGF1‐R)/PI3K/AKT and ERBB2/ERK/CREB signaling in the control of FOXA1 expression in BC cells [37, 38], as well as in the ability of FOXA1 to control IGF‐1R expression [38], has been previously reported. Therefore, the effect of these kinases on FOXA1 expression and activity on the specific enhancer in ESR1 promoter was analyzed by treating cells with two ERBB2 inhibitors (AG‐879, AG‐825), two IGF1‐R inhibitors (NVP AEW541‐NVP; PPP), and one AKT (triciribine‐Tric), ERK (PD 98059‐PD), and CREB (KG 501‐Kg) inhibitor for 48 h in *in‐cell* western blotting [23] and luciferase assays. As shown in Fig. S4A,B, the inhibition of IGF1‐R and AKT reduced FOXA1 expression and activity, thus suggesting that the IGF‐1R/AKT/FOXA1/ERα pathway could be active also in MCF‐7 cells. Accordingly, both depletion of FOXA1, and the chemical inhibition of IGF1‐R and AKT by MK‐2206, an AKT inhibitor in clinical trials for the treatment of breast tumors [39], in MCF‐7 cells all reduced the expression levels of each protein in the pathway (Figs S4C and S5A–C). Therefore, the IGF1‐R/AKT pathway controls FOXA1 and ERα expression and FOXA1 displays a feedback regulation of IGF1‐R expression in MCF‐7 cells.

**Fig. 3:**
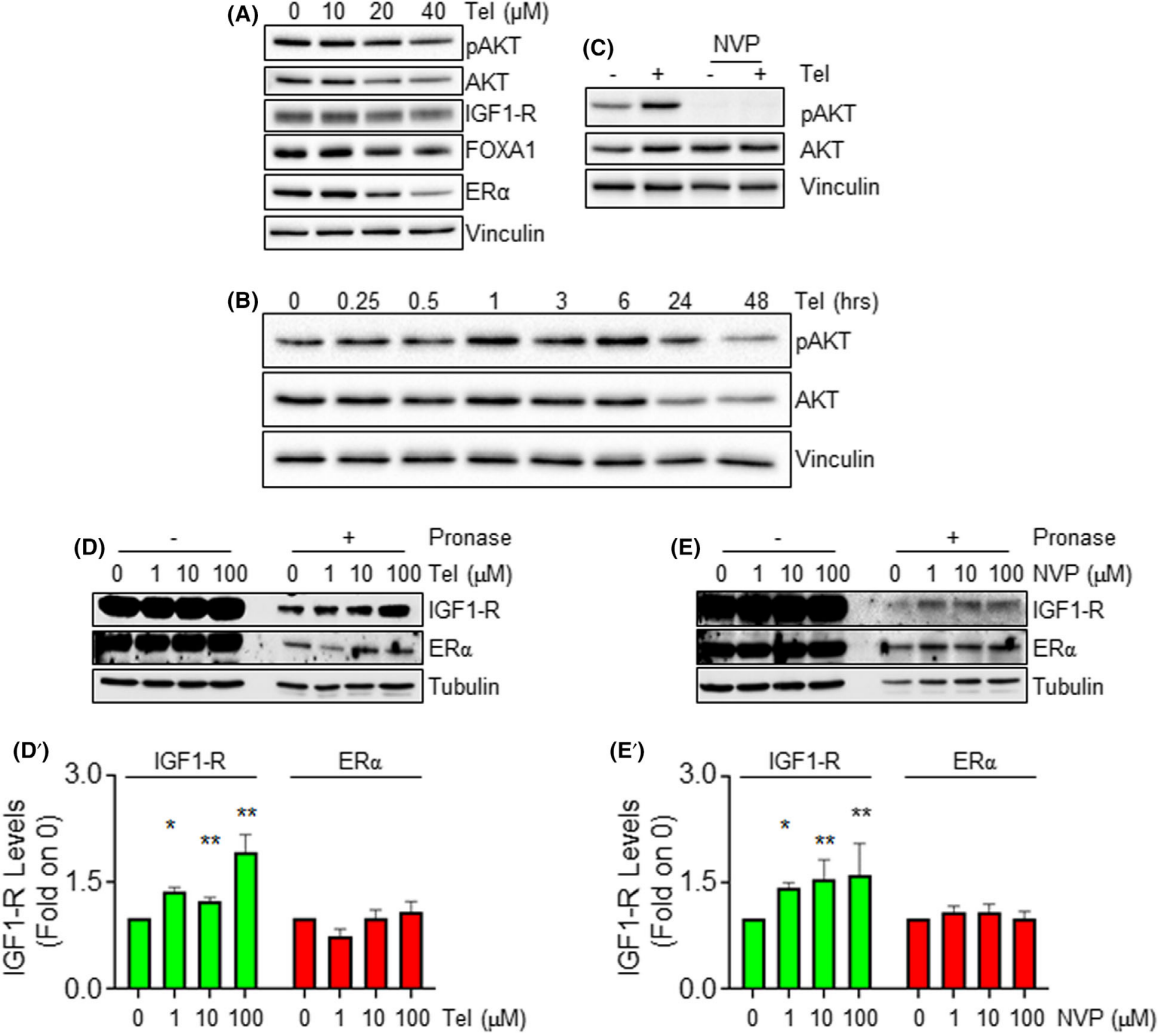
IGF1‐R/AKT/FOXA1 involvement in telaprevir mechanism of action in MCF‐7 cells. (A) Western blotting analyses of pAKT, AKT, IGF1‐R, FOXA1, ERα and vinculin protein levels in MCF‐7 cells treated with the indicated doses of Tel for 48 h. Densitometric analysis is shown in Fig. S3C. *n* = 3 for pAKT and IGF‐1R, *n* = 4 for FOXA1, *n* = 5 for ERα. (B) Western blotting analyses of pAKT, AKT and vinculin protein levels in MCF‐7 cells treated with telaprevir (Tel ‐ 20 μm) at the indicated time points. Densitometric analysis and number of replicates are shown in Fig. S3D. *n* = 3 for 0.25 and 0.5 h, *n* = 4 for 1 and 3 h, *n* = 11 for 6 h, *n* = 6 for 24 and 48 h. (C) Western blotting analyses of pAKT, AKT and vinculin protein levels in MCF‐7 cells pretreated with NVP AEW541 (NVP 1 μm) for 6 h and then treated with Tel (20 μm‐6 h). Densitometric analysis and number of replicates are shown in Fig. S3E. *n* = 6 (D, E, D’, E’) Western blotting analyses and relative densitometric analyses of IGF1‐R, ERα and vinculin protein levels in MCF‐7 cell lysates incubated with increasing doses of telaprevir (Tel 1–100 μm) and NVP AEW541 (NVP 1–100 μm) in the presence or absence of pronase (1 : 1000). Data are the mean ± standard deviation *n* = 3. ** (*P*‐value < 0.01) or * (*P*‐value < 0.05) indicates significant differences with respect to the control sample (0) calculated with one‐way ANOVA followed by the Tukey post‐test. [Colour figure can be viewed at wileyonlinelibrary.com]

Because Tel reduces FOXA1 and ERα intracellular levels in MCF‐7 cells (Fig. 2), Tel could engage the IGF‐1R/AKT pathway. Remarkably, 48 h of the antiviral treatment induced a dose‐dependent reduction in AKT levels and activation as well as in IGF1‐R, FOXA1, and ERα expression in MCF‐7 cells (Fig. 3A and Fig. S3C), thus suggesting IGF1‐R and AKT as a potential upstream regulator of Tel action on FOXA1 and ERα intracellular levels. Furthermore, since Tel mimics the effect of the IGF1‐R inhibitor on the IGF1‐R/AKT/FOXA1/ERα pathway (Figs S4C and S5C), we hypothesized that the antiviral could act as an IGF1‐R inhibitor. To test this hypothesis, Tel impact on IGF‐dependent AKT activation (pAKT) was analyzed in MCF‐7 cells. Cells were pretreated with Tel (20 μm) and NVP (1 μm) for 1, 3, and 6 h before IGF (100 ng·mL^−1^; 15 min) administration. As shown in (Figs S4D and S5D), Tel did not prevent IGF‐dependent AKT activation, while, surprisingly, it slightly increased AKT phosphorylation. Prompted by this observation, MCF‐7 cells were treated with Tel for different time points and AKT phosphorylation was measured. Tel triggers AKT activation, which reaches a peak after 6–24 h of administration (Fig. 3B and Fig. S3D).

Next, the possibility that IGF1‐R mediates Tel‐induced AKT activation was evaluated. Therefore, the effect of NVP on Tel‐dependent AKT phosphorylation was analyzed. As shown in Fig. 3C and Fig. S3E, NVP prevents AKT phosphorylation induced by Tel, thus suggesting the involvement of IGF1‐R in Tel‐dependent AKT activation. As expected, NVP administration to MCF‐7 cells efficiently inhibited IGF‐induced IGF1‐R phosphorylation (Fig. S3F,F’). To further demonstrate that Tel‐induced AKT activation is specific for IGF1‐R, MCF‐7 cells were pretreated with an EGF‐R inhibitor (i.e., gefitinib‐Gef) before Tel administration. Remarkably, Gef did not affect the ability of Tel to induce AKT phosphorylation and NVP pretreatment of MCF‐7 cells prevented IGF‐ but not EGF‐induced AKT phosphorylation while Gef administration blocked EGF‐ but not IGF‐induced AKT phosphorylation (Fig. S6).

Altogether these data demonstrate Tel activates AKT in MCF‐7 cells through the involvement of IGF1‐R.

### 3.4 Identification of IGF1‐R as a possible telaprevir molecular target in MCF‐7 cells

Tel is an antiviral agent targeting the NS3/4A serine protease of the hepatitis C virus [18] and its molecular target in BC cells is unknown. However, the results described above suggest IGF1‐R as a potential Tel binding protein in MCF‐7 cells. To assess this issue, drug affinity responsive target stability (DARTS) analyses were performed. DARTS is a simple method for identifying ligand‐protein interaction based on the reduced susceptibility to proteolysis of the target protein upon ligand binding [30, 31]. In turn, MCF‐7 cell lysates were incubated at R.T. for 1 h *in vitro* with increasing doses of Tel or vehicle in the presence or absence of a protease (i.e., pronase), followed by western blotting analyses of IGF1‐R. The experiment was done in parallel with the *in vitro* incubation of the MCF‐7 cell lysates with different doses of NVP, which is a known functional ligand (i.e., inhibitor) of the IGF1‐R and thus a positive control for IGF1‐R binding. In addition, after the reaction, the ERα levels were further measured in both experiments through western blotting analyses as an internal control for a protein, which is not a ligand of both Tel [19] and NVP. As shown in Fig. 3D,E,D’,E’, Tel and NVP preserve IGF1‐R from protease degradation in a dose‐dependent manner, while they do not influence ERα levels.

Remarkably, the increased resistance to pronase‐dependent proteolysis of IGF1‐R in the presence of Tel suggests an interaction between Tel and IGF1‐R, thus indicating that the IGF1‐R could be a Tel molecular target in MCF‐7 cells.

### 3.5 Telaprevir‐induced caspase‐dependent apoptotic cell death involves both IGF1‐R and FOXA1

The data described so far imply the IGF1‐R/AKT pathway in Tel activity on FOXA1 and ERα protein levels and function and correlate FOXA1 with cell sensitivity to Tel antiproliferative effect in breast and non‐breast tumor cell lines. However, there is no evidence as to which type of cell death is induced by Tel. Indeed, subsequent analyses were performed to identify which type of cell death is induced by the antiviral. For this purpose, apoptosis was assessed in MCF‐7 cells treated with different doses of Tel for 48 h, and both PARP cleavage, DNA fragmentation, and apoptotic nuclei were detected by western blotting, flow cytometry, and immunofluorescent analyses, respectively. Staurosporine (STS), a known apoptosis activator, was also used as the internal control.

**Fig. 4:**
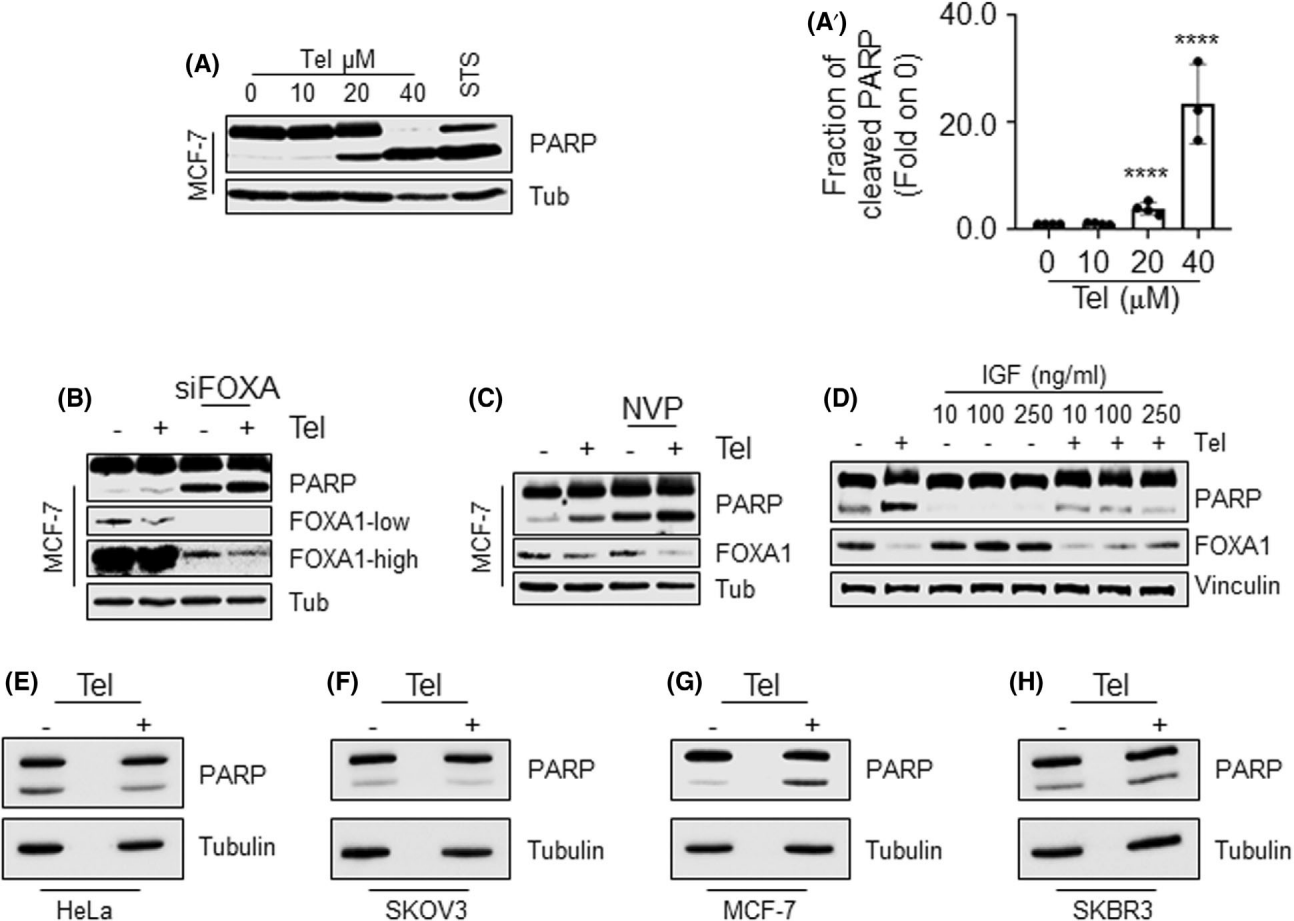
Analysis of telaprevir‐induced apoptosis. (A) Western blotting and (A’) relative densitometric analyses of cleaved PARP and tubulin (Tub) in MCF‐7 cells treated with different doses of telaprevir (Tel 10–40 μm) for 48 h and staurosporine (STS 100 nm) for 24 h. Data are the mean ± standard deviation with *P*‐value < 0.0001. *n* = 4 for all samples but for 40 μm where *n* = 3. **** indicates significant differences with respect to the control sample (0) calculated with one‐way ANOVA followed by the Tukey post‐test. Western blotting analyses of telaprevir (Tel) effect on cleaved PARP, FOXA1 and tubulin (Tub) levels in the presence of FOXA1 siRNA (B) and NVP (C). Cells were transfected with FOXA1 siRNA and treated with 1 μm NVP before 48 h of 20 μm Tel treatment. (D) Western blotting analyses of cleaved PARP, FOXA1 and vinculin expression in MCF‐7 cells pretreated with different doses of IGF (10–250 ng·mL^−1^) for 1 h before 48 h of 20 μm Tel treatment. Densitometric analyses of panel (B) (C) and (D) are shown in Fig. S8C–H. *n* = 4 for (B) (C) and (D), *n* = 3 for FOXA1 levels in panel D. (E–H) Western blotting analyses of cleaved PARP and tubulin in HeLa, SKOV3, MCF‐7, and SKBR3 cells treated with telaprevir (Tel 20 μm) for 48 h. Densitometric analysis are shown in Fig. S9B. *n* = 3 for all the cell lines and *n* = 4 for SKOV3 cells.

The results indicate that Tel induced PARP cleavage (Fig. 4A,A’), increased sub‐diploid cell population, and determined DNA condensation and fragmentation after 48 h in a dose‐dependent manner (Fig. S7A,A’,B). Notably, 24 h Tel administration to MCF‐7 cells induced in a dose‐dependent manner the activation of caspase 9 (Fig. S7C). Therefore, these data indicate that Tel induces cell death by a caspase‐dependent apoptosis in MCF‐7 cells.

The mechanism underlying Tel‐dependent apoptosis was next analyzed, and the involvement of the IGF1‐R/AKT/FOXA1 pathway was tested. To this purpose, MCF‐7 cells were treated with different doses of either the IGF1‐R and the AKT inhibitor (i.e., NVP and MK‐2206, respectively) or were depleted by FOXA1 through siRNA for 48 h and the PARP status was detected. Both IGF1‐R and AKT inhibition, as well as the reduction in FOXA1 levels (Fig. 4B and Fig. S8A,A’,B,B’), induced the cleavage of PARP in a dose‐dependent manner. Notably, this effect phenocopies that of Tel, thus implicating the IGF1‐R/AKT/FOXA1 pathway in the ability of Tel to induce apoptosis.

To prove this observation, MCF‐7 cells were treated with Tel for 48 h both either in the presence of the IGF1‐R inhibitor NVP or the siRNA‐mediated reduction in FOXA1 intracellular levels and PARP levels were measured. Interestingly, co‐treatment of MCF‐7 cells with Tel and either NVP or siRNA‐mediated FOXA1 depletion further increased the amount of PARP cleavage with respect to Tel, NVP, or siRNA‐mediated FOXA1 depletion treatment alone (Fig. 4B,C and Fig. S8C–F).

Therefore, we reasoned that the observed Tel additive effect on PARP cleavage could be because Tel continues to influence the IGF1‐R/AKT/FOXA1 pathway also in the presence of IGF1‐R inhibition or under conditions of FOXA1 protein down‐modulation. In turn, we measured the levels of FOXA1 in the same above‐described experimental conditions. As expected, both Tel, NVP, and siRNA against FOXA1 gene reduced FOXA1 levels in MCF‐7 cells; furthermore, Tel additionally reduced FOXA1 levels in the presence of both NVP treatment and siRNA‐mediated FOXA1 depletion in MCF‐7 cells (Fig. 4B,C and Fig. S8C–F). Overall, these findings strongly suggest that Tel‐induced apoptosis occurs through the inhibition of the IGF1‐R/AKT/FOXA1 pathway.

To support this observation, we reasoned that an activator of the IGF1‐R should rescue the Tel‐induced apoptotic effect. Therefore, the effect of IGF, which binds and activates IGF1‐R, on the ability of Tel to induce PARP cleavage was evaluated. The MCF‐7 cells were pretreated with increasing doses of IGF (from 10 to 250 ng·mL^−1^) for 1 h before 48 h of Tel treatment, and the levels of cleaved PARP and FOXA1 were analyzed. IGF prevents Tel‐induced PARP cleavage and FOXA1 reduction in a dose‐dependent manner (Fig. 4D and Fig. S8G,H).

To further demonstrate that the Tel‐induced apoptotic effect requires both IGF1‐R and FOXA1, we next measured the PARP levels in four different cellular models (i.e., Hela, SKOV3, MCF‐7, and SKBR3 cells), which differentially express or co‐express both IGF1‐R and FOXA1 (Fig. S9A) and display different sensitivity to Tel antiproliferative effects (Fig. 1), treated with Tel for 48 h. Results show that Tel induced the PARP cleavage only in MCF‐7 cells (Fig. 4E–H and Fig. S9B). Therefore, these results indicate that both IGF1‐R and FOXA1 proteins are required for Tel‐induced apoptotic effect.

Overall, these data demonstrate that Tel induces caspase‐dependent apoptosis via the engagement of the IGF1‐R/AKT/FOXA1 pathway.

### 3.6 Cell sensitivity to telaprevir is directly correlated with the FOXA1/IGF1‐R ratio

Although the reported data indicate that Tel‐dependent apoptotic effects require the presence of both IGF1‐R and FOXA1, we surprisingly observed that this antiviral does not induce the PARP cleavage in SKBR3 cells, which display a high sensitivity to Tel antiproliferative effect (Fig. 1).

**Fig. 5:**
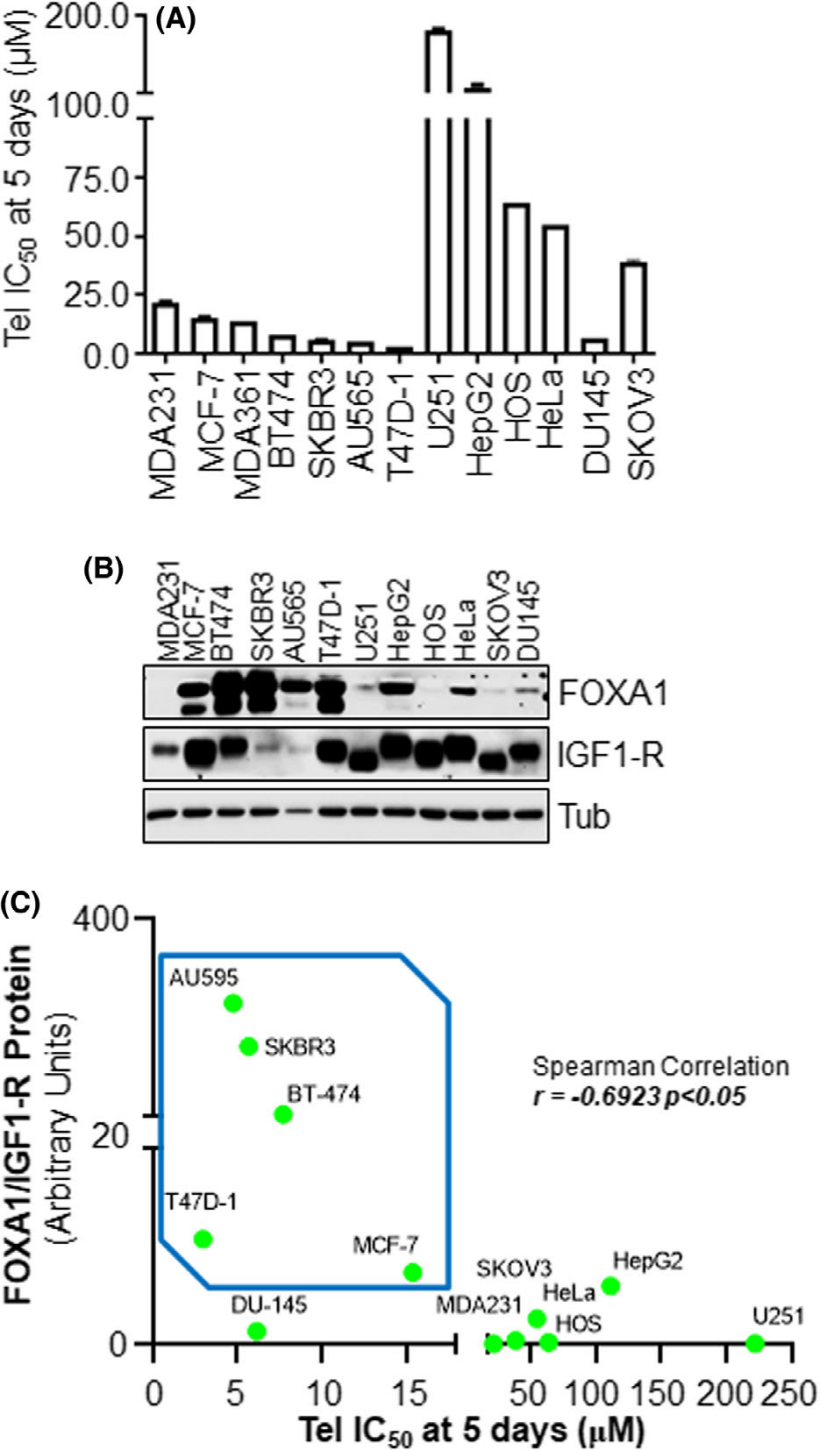
Telaprevir antiproliferative effect is correlated to the ratio of FOXA1/IGF1‐R expression. (A) Bar graph of telaprevir (Tel) inhibition concentration 50 (IC_50_‐μm) value measured in the indicated cancer cell lines at 5 days. Data are the means ± the SD *n* = 3. (B) Western blotting analyses of FOXA1, IGF1‐R and vinculin protein levels in the indicated cancer cell lines. This blot has been performed twice. (C) The scatter plot of Spearman correlation analyses for the ratio of FOXA1/IGF1‐R protein levels and Tel IC_50_ (μm) on the proliferation of the above‐mentioned cancer cell lines. [Colour figure can be viewed at wileyonlinelibrary.com]

Therefore, to understand this paradox, we measured the sensitivity to the Tel effect of 12 different cancer cell lines by measuring the Tel IC_50_ on cell proliferation at 5 days (Fig. 5A). The Tel IC_50_ was then compared with both IGF1‐R and FOXA1 protein expression levels in the same cell lines (Fig. 5B). Spearman correlation index was calculated considering the potential correlation between both the Tel IC_50_‐FOXA1 levels, the Tel IC_50_‐IGF1‐R levels, and the Tel IC_50_‐FOXA1/IGF1‐R levels ratio. Interestingly, no correlation between cell sensitivity to Tel antiproliferative effect and the levels of either IGF1‐R or FOXA1 was found (data not shown). In contrast, a significant correlation between the sensitivity to Tel antiproliferative effect and the ratio of FOXA1/IGF1‐R expression was evidenced (Fig. 5C). These observations indicate that the higher the ratio of FOXA1/IGF1‐R protein levels the higher sensitivity of cell lines to Tel antiproliferative effect.

Overall, these data demonstrate that Tel antiproliferative effects are linearly correlated with increasing amount of the ratio between FOXA1 and IGF1‐R protein expression.

### 3.7 Preclinical evaluation of telaprevir as a novel drug for the treatment of primary and metastatic breast cancer

**Fig. 6:**
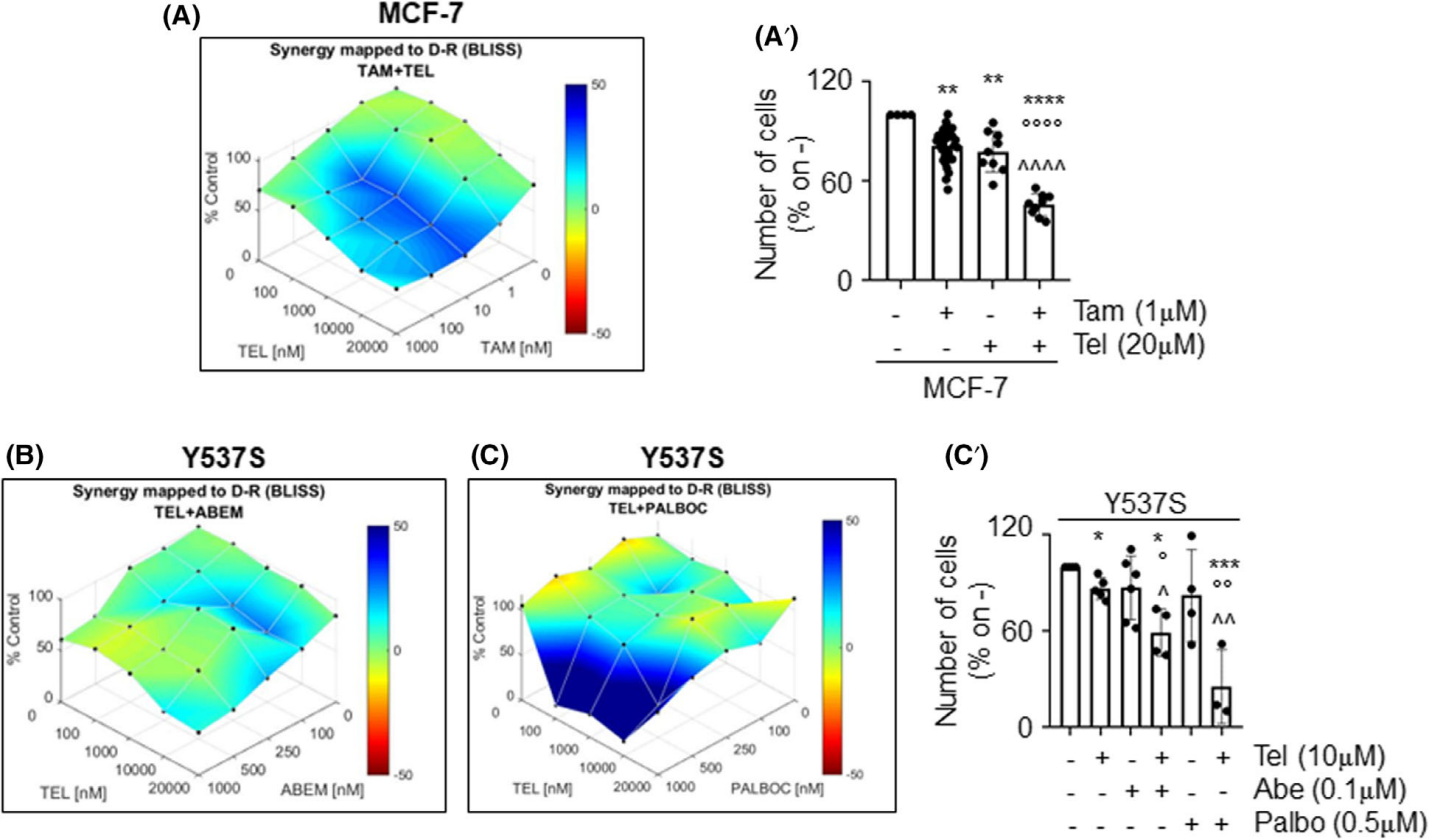
Preclinical assessment of Tel effect in breast cancer treatment. (A) Synergy distribution mapped to the dose–response surface, determined by Bliss interaction model and generated by combenefit software. Telaprevir (Tel, from 100 to 20 000 nm) and 4OH‐tamoxifen (Tam, from 1 to 1000 nm) were added separately or in combination to MCF‐7 cells for 12 days. Data were normalized as % of the control. (A’) The effect on cell number of Tel alone or in combination with Tam. Data are the mean ± standard deviation with a *P*‐value < 0.01 (**), 0.0001 (****, °°°°, ^^^^). * Indicates significant differences calculated with one‐way ANOVA followed by the Tukey post‐test with respect to the control sample (−−). *n* = 36 for Tam alone, *n* = 9 for Tel alone Tam + Tel. ° Indicates significant differences calculated with one‐way ANOVA followed by the Tukey post‐test with respect to Tam‐treated sample (+−). ^ indicates significant differences calculated with Student *t*‐test with respect to Tel‐treated sample (−+). Sinergy map of Y537S‐ERα‐expressing MCF‐7 cells (Y537S) treated with Tel (from 100 to 20 000 nm) alone or in combination with abemaciclib (Abe, from 100 to 1000 nm) (B) or palbociclib (Palbo, from 100 to 1000 nm) (C) for 12 days. Data were normalized as % of the control. (C’) The effect on cell number of Tel alone or in combination with Abe or Palbo in Y537S. Data are the mean ± standard deviation with a *P*‐value < 0.1 (*, °, ^), 0.01 (°°, ^^), 0.001 (***). * indicates significant differences calculated with one‐way ANOVA followed by the Tukey post‐test with respect to the control sample (−−−), ° indicates significant differences calculated with one‐way ANOVA followed by the Tukey post‐test with respect to Tel‐treated sample (+−−), ^ indicates significant differences calculated with one‐way ANOVA followed by the Tukey post‐test with respect to Abe (−+−)‐ or Palbo (−−+)‐treated sample. *n* = 5 for Tel alone, *n* = 6 for abe alone, *n* = 4 for palbo alone, *n* = 4 for Tel + abe and *n* = 3 for Tel + palbo. [Colour figure can be viewed at wileyonlinelibrary.com]

E2 signaling sustains BC development, growth, and survival and 70% of BC cases express ERα, which acts as the main driver of E2 mitogenic stimuli [40]. Remarkably, Tel displays inhibitory activity against E2:ERα signaling similar to that of ET drugs already used in the treatment of BC [19]. Therefore, the possibility that Tel exhibits a synergistic effect with drugs used for the treatment of primary and metastatic BC was evaluated. For this purpose, MCF‐7 cells were exposed to increasing doses of both the selective estrogen receptor modulator prototype 4OH‐tamoxifen (Tam—from 1 to 1000 nm) and Tel (from 100 to 20 000 nm), administered either alone or in combination, for 12 days. As shown in Fig. 6A,A’, Tel exhibits synergism with Tam in preventing cell proliferation [combination index (CI) < 1, CI_Tam + Tel_MCF‐7_ = 0.047 ± 0.037].

Moreover, the cyclin‐dependent kinase (CDK) 4/6 inhibitors have recently been introduced in the treatment of metastatic BC [41]. Therefore, combination analyses between Tel and the CDK4/6 inhibitors abemaciclib (Abe) and palbociclib (Palbo) were performed in a cellular model mimicking the metastatic breast cancer expressing the ERα mutated in the Y residue 537 to S (i.e., Y537S cells) [42]. The results reported in Fig. 6B,C,C’ indicate a synergism between both Tel and Abe and Tel and Palbo in Y537S cells [combination index (CI) < 1, CI_Abe + Tel_Y537S_ = 0.68 ± 0.36; CI_Palbo + Tel_Y537S_ = 0.47 ± 0.53].

Overall, these findings suggest that Tel could be used as an adjuvant drug in the treatment of primary and metastatic BC.

## 4 Discussion

Here, we aimed to dissect the molecular mechanism of telaprevir (Tel) effect on the control of ERα levels and function and cell proliferation in breast cancer (BC) cells. Notably, we made two key discoveries to attain this goal.

First, we previously performed an array analysis of ERα signaling [19] and we found FOXA1 among the genes to be down‐modulated by Tel. FOXA1 is a pioneer factor able to bind condensed chromatin and to modify chromatin structure to euchromatin conditions, thus enabling the binding of other transcription factors, such as ERα [35, 36]. In turn, FOXA1 regulates *ESR1* transcription and correlates with ERα expression in BC [36]. Thus, since Tel controls ERα expression at the transcriptional level [19] and FOXA1 protein downregulation reduces receptor levels in BC cells [35], we hypothesized that Tel could affect receptor expression by influencing FOXA1. Indeed, the results showed that Tel reduces FOXA1 and ERα levels in cell lines that express both proteins (i.e., MCF‐7 and BT‐474 cells) as well as FOXA1 expression in SKBR3 and AU565 cells, which express only FOXA1. Conversely, Tel does not affect ERα levels in cells that do not express FOXA1, such as SKOV3 cells. Moreover, we evaluated Tel direct impact on FOXA1‐dependent control of ERα expression in MCF‐7 ESR1‐NLuc cells and we noticed that Tel not only decreases FOXA1 levels but also prevents FOXA1 activity on its specific enhancer in the *ESR1* promoter. Furthermore, FOXA1 plays an important role in the control of ERα transcriptional activity in BC cells [43, 44]. Accordingly, siRNA‐mediated FOXA1 depletion significantly reduces ERα transcriptional activity in MCF‐7 ERE‐NLuc cells. In addition, we observed a reduction of pS2 expression in the absence of FOXA1 expression in MCF‐7 cells, which is consistent with the decrease in ERα binding to TFF1 (i.e., pS2) gene promoter following the ablation of FOXA1 expression [45]. Because Tel prevents ERα transcriptional activity [19] and affects FOXA1 levels and action, we investigated FOXA1 role in Tel impact on ERα nuclear function. In turn, Tel ability to prevent ERα transcriptional activity and pS2 expression is impaired when FOXA1 expression is down‐modulated. Therefore, FOXA1 is an intermediary factor of Tel action on ERα levels and function and Tel‐dependent reduction in FOXA1 levels and activity is necessary for the decrease in ERα levels and function induced by the antiviral.

Second, we evaluated Tel antiproliferative effect on a panel of nontransformed (i.e., human fibroblast and MCF10a cells), breast tumor (i.e., MDA‐MB‐231, MCF‐7, T47D‐1, BT‐474, SKBR3, and AU565 cells) and non‐breast tumor (i.e., HeLa, U251, SKOV3, and DU145 cells) cell lines. We calculated the Tel inhibition concentration 50 (IC_50_) values required to prevent cell proliferation and we considered sensitive to Tel antiproliferative effect the cell lines characterized by an IC_50_ lower than 20 μm. Remarkably, Tel does not affect the proliferation of nontransformed cell lines, while the antiviral inhibits the proliferation of all the breast tumor cell lines and the prostate carcinoma cell line DU145 cells. It is noteworthy that the common factor of these cell lines sensitive to the Tel antiproliferative effect is the expression of FOXA1 mRNA. Accordingly, FOXA1 plays an important role both in breast and prostate tumorigenesis [46]. In turn, the presented results indicate that FOXA1 is upstream to ERα in the control of cell proliferation and demonstrate that FOXA1 is required for Tel ability to prevent cell proliferation. Indeed, siRNA‐mediated FOXA1 down‐modulation decreases Tel antiproliferative effect in FOXA1‐expressing SKBR3 cells (IC_50_ lower than 20 μm), while FOXA1 overexpression in SKOV3 cells (IC_50_ higher than 20 μm), which do not express the pioneer factor, enhances Tel antiproliferative effect.

Overall, these observations establish FOXA1 as one of the upstream regulators of Tel action on the control of ERα levels and cell proliferation in BC cells.

The subsequent aim was the identification of the other members of the Tel mechanism of action, as well as the Tel molecular target in BC cells. Thus, consistent with the role for insulin‐like growth factor 1 receptor (IGF1‐R)/PI3K/AKT and ERBB2/ERK/CREB signaling in the control of FOXA1 expression in BC cells [37, 38], we performed a small‐scale screening of kinase inhibitors in MCF‐7 cells. The screen revealed that IGF1‐R and AKT inhibitors mimic Tel effect on both FOXA1 levels and activity, AKT levels, and phosphorylation, IGF1‐R, and ERα levels. Therefore, we tested the possibility that Tel acts as an IGF1‐R inhibitor. However, Tel does not inhibit IGF1‐R‐mediated IGF‐induced AKT activation. Nonetheless, we observed that Tel activates AKT in 6–24 h in MCF‐7 cells. Notably, Tel‐dependent AKT activation is mediated by IGF1‐R. Indeed, it is prevented by NVP in MCF‐7 cells. Therefore, we analyzed the possibility that IGF1‐R could be a potential candidate as Tel molecular target in BC cells. To test this hypothesis, we performed a drug affinity responsive target stability (DARTS) assay [30, 31] and, surprisingly, we noticed that IGF1‐R susceptibility to protease degradation is reduced in the presence of Tel. Although we did not undertake a binding assay to confirm Tel binding to IGF1‐R, these findings strongly suggest an interaction between IGF1‐R and Tel and define a signal transduction pathway (i.e., IGF1‐R/AKT/FOXA1) for Tel action in MCF‐7 cells.

Moreover, the obtained results indicate that the above‐mentioned pathway mediates Tel‐induced caspase‐dependent apoptosis. Indeed, either IGF1‐R inhibition, AKT inhibition, and siRNA‐mediated FOXA1 depletion phenocopy Tel‐dependent induction of PARP cleavage. Notably, the results suggest that Tel‐dependent inhibition of IGF1‐R/AKT/FOXA1 pathway is required for Tel apoptotic effect, and the analyses of Tel‐dependent PARP cleavage in four different cellular models (i.e., Hela, SKOV3, MCF‐7, and SKBR3 cells) that differentially express IGF1‐R and/or FOXA1 indicate that both proteins are required for Tel‐induced PARP cleavage. Accordingly, the antiviral induces apoptosis exclusively in MCF‐7 cells, which express high levels of both IGF1‐R and FOXA1 proteins. However, this result encloses a contradiction since Tel does not induce PARP cleavage in SKBR3 cells, which exclusively express FOXA1 and exhibit high sensitivity to Tel antiproliferative effect. To solve this paradox, we compared the Tel IC_50_ on the proliferation of different cell lines with the expression levels of IGF1‐R and FOXA1 proteins. Remarkably, we observed that a correlation exists between Tel antiproliferative effect and the FOXA1/IGF1‐R ratio. These data demonstrate that the higher the ratio of FOXA1/IGF1‐R expression the higher the sensitivity to the Tel antiproliferative effect.

Notably, about 50% of BC express the activated form of IGF1‐R, which sustains tumor growth, survival, and motility [47]. In addition, FOXA1 expression is associated with luminal A subtype of BC [46] and its overexpression mediates endocrine resistance in metastatic BC by inducing the redistribution of ERα binding sites, which alter the expression of ERα target genes [48]. Therefore, targeting FOXA1 could provide an appealing strategy for BC treatment. In this respect, the discovery that Tel prevents the proliferation of different BC cell lines acting as a model of primary and metastatic BC, while the proliferation of nontransformed cell lines is not affected by the antiviral together with the ability of Tel to synergistically work with both ET drugs (i.e., tamoxifen) and with the drugs used for the treatment of metastatic BC (e.g., abemaciclib and palbociclib) strongly indicate that Tel could be repurposed for the treatment of ERα‐expressing BCs. Moreover, our data further suggest that this antiviral could be effective for the treatment of those cancers, which remain addicted to the IGF1‐R/AKT/FOXA1 pathway.

Finally, it is important to point out that the doses we used in this work (i.e., 10–20 μm) are in the range of the plasma concentration of Tel reached after administration of the antiviral at therapeutic doses [49]. Indeed, the maximum plasma concentration reached after Tel administration is 5.4 μm at a steady state [50].

## 5 Conclusions

The molecular characterization of Tel action discloses a novel ERα pathway (i.e., IGF1‐R/AKT/FOXA1) influencing the control of ERα levels and BC cell proliferation and finally suggests Tel repurposing as a new approach for the treatment of ERα‐expressing BCs.

## Conflict of interest

The authors declare no conflict of interest.

## Author contributions

SB performed most of the experiments. SL performed apoptosis and growth curve analyses. SP and MC performed signaling studies. FA designed the research, analyzed the data, prepared the figures, and wrote the text.

### Peer Review

The peer review history for this article is available at https://publons.com/publon/10.1002/1878‐0261.13303.

## Supporting information


**Fig. S1.** Controls for FOXA1 siRNA and overexpression.

**Fig. S2.** Controls for siRNA‐mediated effects of FOXA1.

**Fig. S3.** Histograms relative to the western blots shown in main figures.

**Fig. S4.** Tel impact on IGF1‐R/AKT/FOXA1 signaling pathway.

**Fig. S5.** Histograms relative to the western blots shown in Supplementary figures.

**Fig. S6.** Specificity controls for the IGF1‐R and EGF‐R inhibitors.

**Fig. S7.** Telaprevir‐induced apoptosis.

**Fig. S8.** Impact of IGF1‐R/AKT/FOXA1 pathway in apoptosis induction.

**Fig. S9.** Telaprevir effect of apoptosis induction in different cell lines.

**Appendix S1.** Legends.

**Appendix S2.** Figures.

## Data Availability

The data presented in this study are available in supplementary material here.
