# Supplementary figures and images for: The antiviral drug telaprevir induces cell death by reducing FOXA1 expression in estrogen receptor α (ERα)‐positive breast cancer cells
Source: Mol Oncol. 2022 Sep 3;16(19):3568–84. doi: 10.1002/1878-0261.13303 (PMC9533686; doi:10.1002/1878-0261.13303)

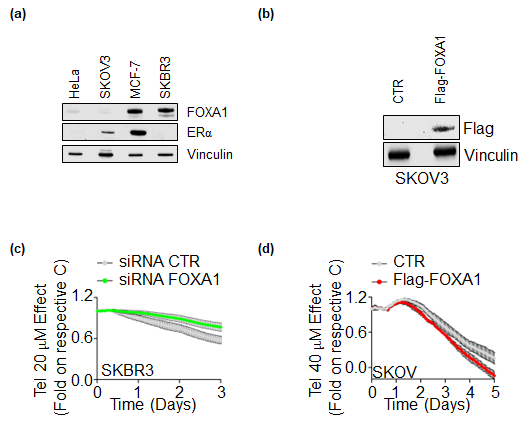

Supplement: Supplementary file 1 — Fig. S1. Controls for FOXA1 siRNA and overexpression. [file MOL2-16-3568-s004.tif]

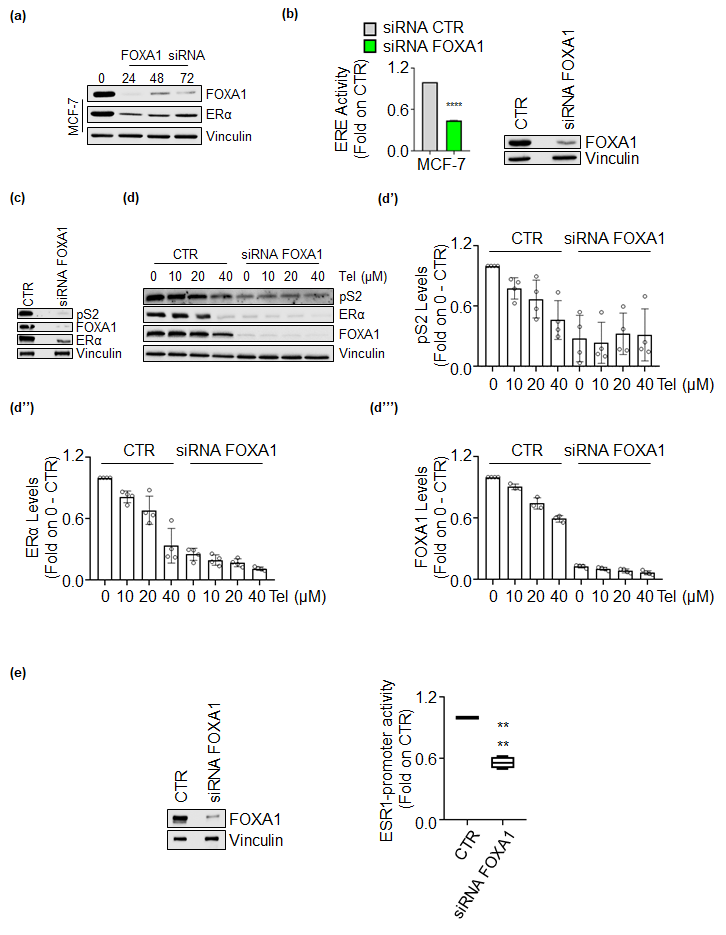

Supplement: Supplementary file 2 — Fig. S2. Controls for siRNA‐mediated effects of FOXA1. [file MOL2-16-3568-s011.tif]

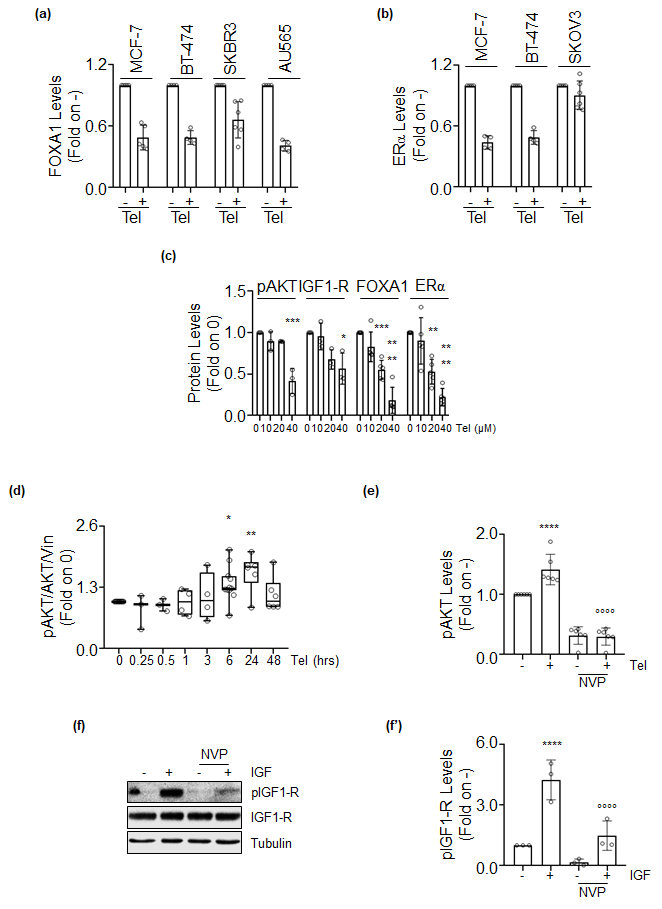

Supplement: Supplementary file 3 — Fig. S3. Histograms relative to the western blots shown in main figures. [file MOL2-16-3568-s005.tif]

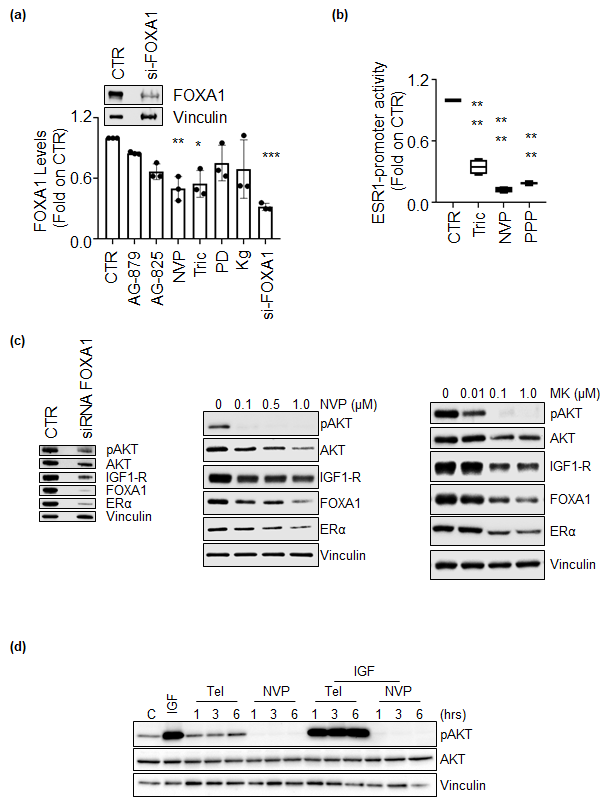

Supplement: Supplementary file 4 — Fig. S4. Tel impact on IGF1‐R/AKT/FOXA1 signaling pathway. [file MOL2-16-3568-s008.tif]

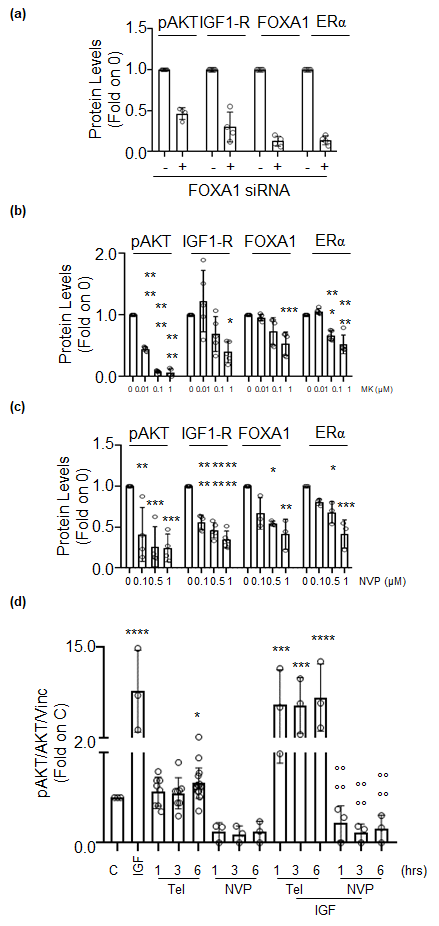

Supplement: Supplementary file 5 — Fig. S5. Histograms relative to the western blots shown in Supplementary figures. [file MOL2-16-3568-s003.tif]

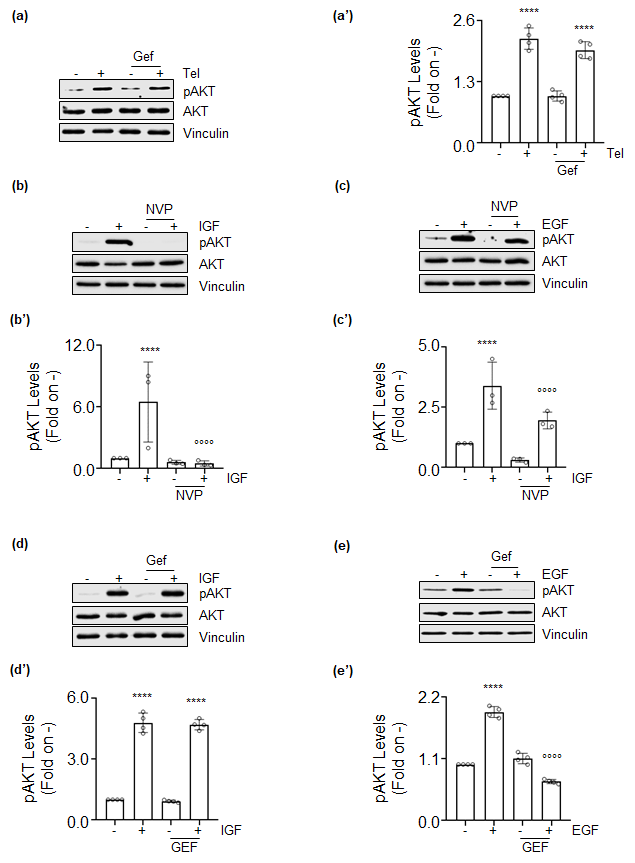

Supplement: Supplementary file 6 — Fig. S6. Specificity controls for the IGF1‐R and EGF‐R inhibitors. [file MOL2-16-3568-s001.tif]

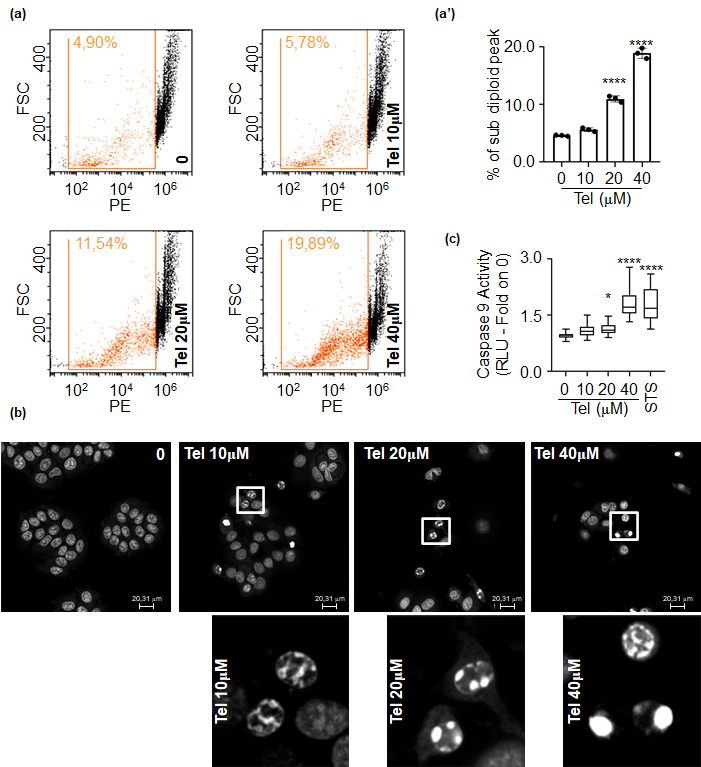

Supplement: Supplementary file 7 — Fig. S7. Telaprevir‐induced apoptosis. [file MOL2-16-3568-s009.tif]

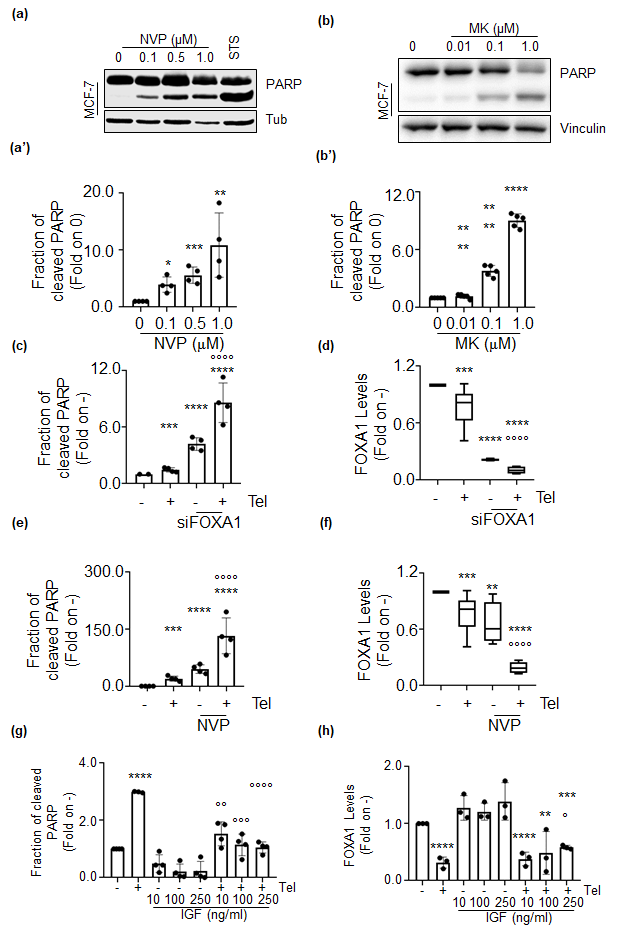

Supplement: Supplementary file 8 — Fig. S8. Impact of IGF1‐R/AKT/FOXA1 pathway in apoptosis induction. [file MOL2-16-3568-s007.tif]

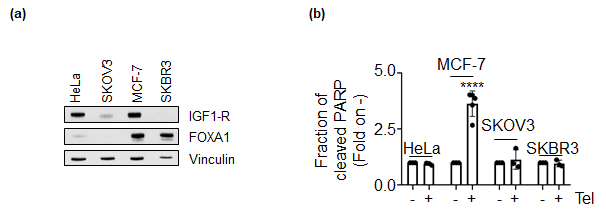

Supplement: Supplementary file 9 — Fig. S9. Telaprevir effect of apoptosis induction in different cell lines. [file MOL2-16-3568-s010.tif]
