# Supplementary material for: The antiviral drug telaprevir induces cell death by reducing FOXA1 expression in estrogen receptor α (ERα)‐positive breast cancer cells
Source: Mol Oncol. 2022 Sep 3;16(19):3568–84. doi: 10.1002/1878-0261.13303 (PMC9533686; doi:10.1002/1878-0261.13303)
Supplement: Supplementary file 10 — Appendix S1. Legends. [file MOL2-16-3568-s002.docx]

**Supplementary Figure Captions**

**Figure 1. Controls for FOXA1 siRNA and overexpression.**

(a) Western blotting analyses of basal FOXA1, ERα and vinculin protein levels in HeLa, SKOV3, MCF-7 and SKBR3 cells. (b) Western blotting analyses of flag-tagged FOXA1 and vinculin expression levels in SKOV3 cells stably expressing FOXA1 *vs* parental SKOV3 cells. (c) Real-time growth curves analyses in SKBR3 cells treated with Tel 20 µM in the presence or absence of FOXA1 siRNA for the indicated time points. Data are the mean ± standard errors n=8. (d) Real-time growth curve analyses in SKOV3 cells stably expressing FOXA1 *vs* parental SKOV3 cells treated with Tel 40 µM for the indicated time points. Data are the mean ± standard errors n=8.

**Figure 2. Controls for siRNA-medieted effects of FOXA1.**

(a) Western blotting analysis of FOXA1, ERα and vinculin protein levels after 24, 48, and 72 hrs of FOXA1 siRNA depletion in MCF-7 cells. (b) ERE promoter activity in MCF-7 ERE-NLuc cells after 48 hrs of FOXA1 siRNA depletion and Western blotting analyses of FOXA1 and vinculin protein levels in MCF-7 ERE-NLuc cells both in the presence and absence of FOXA1 siRNA. Data are the mean ± standard deviation with a p-value < 0.001 (***) and 0.0001 (****). * Indicates significant differences with respect to the control sample (siRNA CTR) calculated with Student t-test. Western blotting analyses of presenilin 2 (pS2), FOXA1, ERα, and vinculin protein levels in MCF-7 after 48 hrs of FOXA1 siRNA depletion (c) and after 48 hrs of Tel (10-40 µM) treatment in the presence or absence of FOXA1 siRNA (d). Densitometric analyses are reported in panels (d’) for pS2, (d’’) for ERα, and in (d’’’) for FOXA1. (e) The activity of FOXA1 specific enhancer in ESR1 promoter region measured in MCF-7 ESR1-NLuc cells treated in the presence or the absence of FOXA1 siRNA. The experiment was performed twice in a quintupled. (e) Western blotting analyses of FOXA1 protein levels in MCF-7 ESR1-NLuc cells both in the presence and the absence of FOXA1 siRNA. Data are the mean ± standard deviation with a p-value < 0.0001 (****). * Indicates significant differences with respect to the control sample (CTR) calculated with Student t-test. n=3 for panel b, n=4 for panel d’ and d’’, n=3 for panel d’’’ and n=4 for panel e.

**Figure 3. Histograms relative to the Western blots shown in main figures.**

Densitometric analyses relative to Fig. 2a and 2b of FOXA1 (a) and ERα (b) levels in the indicated cell lines treated with telaprevir (Tel 20 µM). (c) Densitometric analyses relative to Fig. 3a of pAKT, IGF1-R, FOXA1 and ERα levels in MCF-7 cells treated with the indicated doses of telaprevir (Tel). Data are the mean ± standard deviation. **** (p-value < 0.0001), *** (p-value < 0.001), ** (p-value < 0.01) or * (p-value < 0.05) indicates significant differences with respect to the control sample (0) calculated with one-way Anova followed by the Tukey post-test. (d) Densitometric analyses relative to Fig. 3b of pAKT levels in MCF-7 cells treated at the indicated time points with telaprevir (Tel 20 µM). Data are the mean ± standard deviation. ** (p-value < 0.01) or * (p-value < 0.05) indicates significant differences with respect to the control sample (0) calculated with one-way Anova followed by the Tukey post-test. (e) Densitometric analyses relative to Fig. 3c of pAKT in MCF-7 cells treated with telaprevir (Tel 20 µM) in the presence or in the absence of NVP AEW541 (NVP). Data are the mean ± standard deviation. **** and °°°° (p-value < 0.0001) indicates significant differences with respect to the control sample (-) and and treated sample (+) calculated with one-way Anova followed by the Tukey post-test. Western blotting (f) and relative densitometric analyses (f’) of pIGF1-R, IGF1-R and tubulin protein levels in MCF-7 cells treated with IGF (100 ng/ml - 2 min) in the presence or in the absence of NVP AEW541 (NVP). Data are the mean ± standard deviation. **** and °°°° (p-value < 0.0001) indicates significant differences with respect to the control sample (-) and treated sample (+) calculated with one-way Anova followed by the Tukey post-test.

**Figure 4. Tel impact on IGF1-R/AKT/FOXA1 signaling pathway.**

(a) Analysis of FOXA1 levels in MCF-7 cells by *in-cell* Western blotting. Cells were treated with 1 µM of AG-879, and AG-825 (i.e., ERBB2 inhibitors), NVP AEW541-NVP (i.e., IGF1-R inhibitor), triciribine-Tric (i.e., AKT inhibitor), PD 98059-PD (i.e., ERK inhibitor), and KG 501-Kg (i.e., CREB inhibitor) for 48 hrs. FOXA1 siRNA was used as the internal control. Data are the mean ± standard deviation. *** (p-value < 0.001), ** (p-value < 0.01) or * (p-value < 0.05) indicates significant differences with respect to the control sample (CTR) calculated with one-way Anova followed by the Tukey post-test. Inset shows the Western blotting analyses of FOXA1 and vinculin expression in the cells treated with FOXA1 siRNA. (b) The activity of FOXA1 specific enhancer in ESR1 promoter region measured in MCF-7 ESR1-NLuc cells treated with 1 µM Tric, 1 µM NVP, and 1 nM PPP (i.e., IGF1-R inhibitor) for 48 hrs. Data are the mean ± standard deviation with a p-value < 0.0001 (****). * Indicates significant differences with respect to the control sample (CTR) calculated with one-way Anova followed by the Tukey post-test. The experiment was performed twice in a quadruplicate. (c) Western blotting analyses of pAKT, AKT, FOXA1, ERα, IGF1-R and vinculin protein levels in MCF-7 cells treated with FOXA1 siRNA and different concentration of the AKT inhibitor MK-2206 (MK) (0.01 to 1 µM) in clinical trials for breast cancer treatment, and NVP (0.1 to 1 µM) for 48 hrs. Data are the mean ± standard deviation. **** (p-value < 0.0001), *** (p-value < 0.001), ** (p-value < 0.01) or * (p-value < 0.05) indicates significant differences with respect to the control sample calculated with one-way Anova followed by the Tukey post-test. Densitometric analyses are reported in Supplementary Fig. 4a, 4b, and 4c. (d) Western blotting analyses of pAKT, AKT and vinculin protein levels in MCF-7 cells pre-treated with telaprevir (Tel 20 µM) and NVP AEW541 (NVP 1 µM) for 1, 3, and 6 hrs before 15 minutes of IGF (100 ng/ml) administration. Data are the mean ± standard deviation. **** (p-value < 0.0001), *** (p-value < 0.001), or * (p-value < 0.05) indicates significant differences with respect to the control sample (CTR) calculated with one-way Anova followed by the Tukey post-test. Densitometric analyses are reported in Supplementary Fig. 4d. n=3 for panel f’.

**Figure 5. Histograms relative to the Western blots shown in Supplementary figures.**

Densitometric analyses relative to Supplementary Fig. 3c of pAKT, AKT, IGF1-R, FOXA1 and ERα levels in MCF-7 cells treated with FOXA1 siRNA (a), and with the indicated doses of both MK-2206 (MK) (b) and NVP AEW541 (NVP) (c). (d) Densitomentric analyses relative to Supplementary Fig. 3d. n=4 for panel a’, d’ and e’ and n=3 for panel b’ and c’.

**Figure 6. Specificity controls for the IGF1-R and EGF-R inhibitors.**

Western blotting (a-e) and relative densitometric analyses (a’-e’) of pAKT, AKT and vinculin protein levels in MCF-7 cells pre-treated with with gefitinib (Gef) 0.1 µM for 6 hrs and then treated with Tel (20 µM - 6 h) (a, a’), IGF (100 ng/ml-15 min) (d, d’) and EGF (100 ng/ml-5 min) (e, e’). Western blotting analyses of pAKT, AKT and vinculin protein levels in MCF-7 cells pre-treated with NVP AEW541 (NVP 1 µM) for 6 hrs and then treated with IGF (100 ng/ml-15 min) (b, b’) and EGF (100 ng/ml-5 min) (c, c’). Data are the mean ± standard deviation. **** and °°°° (p-value < 0.0001) indicates significant differences with respect to the control sample (-) and treated sample (+) calculated with one-way Anova followed by the Tukey post-test.

**Figure 7. Telaprevir-induced apoptosis**

(a) Flow cytometry dot plot and (a’) bar diagram representing the effect of different doses of Tel (10 to 40 µM-48 hrs) on a sub-diploid fraction in MCF-7 cells. Data are the mean ± standard deviation with a p-value < 0.0001. **** Indicates significant differences with respect to the control sample (0) calculated with one-way Anova followed by the Tukey post-test. (b) Confocal microscopy of nuclear morphology in MCF-7 cells stained with 4′,6-diamidino-2-phenylindole (DAPI) after 48 hrs of Tel (10 to 40 µM) treatment. (c) Caspase 9 activity was measured in MCF-7 cells treated with the indicated doses of telaprevir (Tel) or staurosporine (STS 100 nM) for 24 hrs. Data are the mean ± standard deviation. **** (p-value < 0.0001) and * (p-value < 0.05) indicates significant differences with respect to the control sample (0) calculated with with one-way Anova followed by the Tukey post-test. n=3 for panel a’, n=50 for panel c.

**Figure 8. The impact of IGF1-R/AKT/FOXA1 pathway in apoptosis induction.**

(a, a’, b, b’) Western blotting and relative densitometric analyses of cleaved PARP, and tubulin (Tub) in MCF-7 cells treated with NVP AEW541 (NVP 0.1 to 1 µM), MK-2206 (MK 0.01 to 1 µM) for 48 hrs and staurosporine (STS 100 nM) for 24 hrs. Data are the mean ± standard deviation with p-value <0.05 (*), < 0.01 (**), 0.001 (***) and 0.0001 (****). * Indicates significant differences with respect to the control sample (0) calculated with one-way Anova followed by the Tukey post-test. Densitometric analyses relative to figure 4b and 4c of telaprevir (Tel) effect on cleaved PARP, FOXA1 and tubulin (Tub) levels in the presence of FOXA1 siRNA (c, d) and NVP (e, f). Data are the mean ± standard deviation with p-value < 0.01 (**), 0.001 (***) and 0.0001 (****, °°°°). * Indicates significant differences with respect to the control sample (-); ° indicates significant differences with respect to siRNA FOXA1 or NVP control sample calculated with one-way Anova followed by the Tukey post-test. (g, h) Densitometric analyses relative to figure 4d of cleaved PARP, and FOXA1 expression in MCF-7 cells pre-treated with different doses of IGF (10 to 250 ng/ml) for 1 hour before 48 hrs of 20 µM Tel treatment. Data are the mean ± standard deviation with p-value < 0.1 (°), 0.01 (**-°°), 0.001 (***-°°°) and 0.0001 (****-°°°°). * Indicated significant differences with respect to the control sample (-) calculated with one-way Anova followed by the Tukey post-test; ° indicates significant differences with respect to IGF‐treated samples calculated with one-way Anova followed by the Tukey post-test.

**Figure 9. Telaprevir effect of apoptosis induction in different cell lines.**

(a) Western blotting analyses of IGF1-R, FOXA1 and vinculin expression levels in HeLa, SKOV3, MCF-7, and SKBR3 cells. Densitometric analyses relative to Fig. 4e-4h of cleaved PARP in HeLa, SKOV3, MCF-7, and SKBR3 cells treated with telaprevir (Tel 20 µM) for 48 hrs. Data are the mean ± standard deviation with p-value < 0.0001 (****). * Indicates significant differences with respect to the control sample (-) calculated with Student t-test.
