# Supplementary material for: The antiviral drug telaprevir induces cell death by reducing FOXA1 expression in estrogen receptor α (ERα)‐positive breast cancer cells
Source: Mol Oncol. 2022 Sep 3;16(19):3568–84. doi: 10.1002/1878-0261.13303 (PMC9533686; doi:10.1002/1878-0261.13303)
Supplement: Supplementary file 11 — Appendix S2. Figures. [file MOL2-16-3568-s006.pptx]

## Slide 1
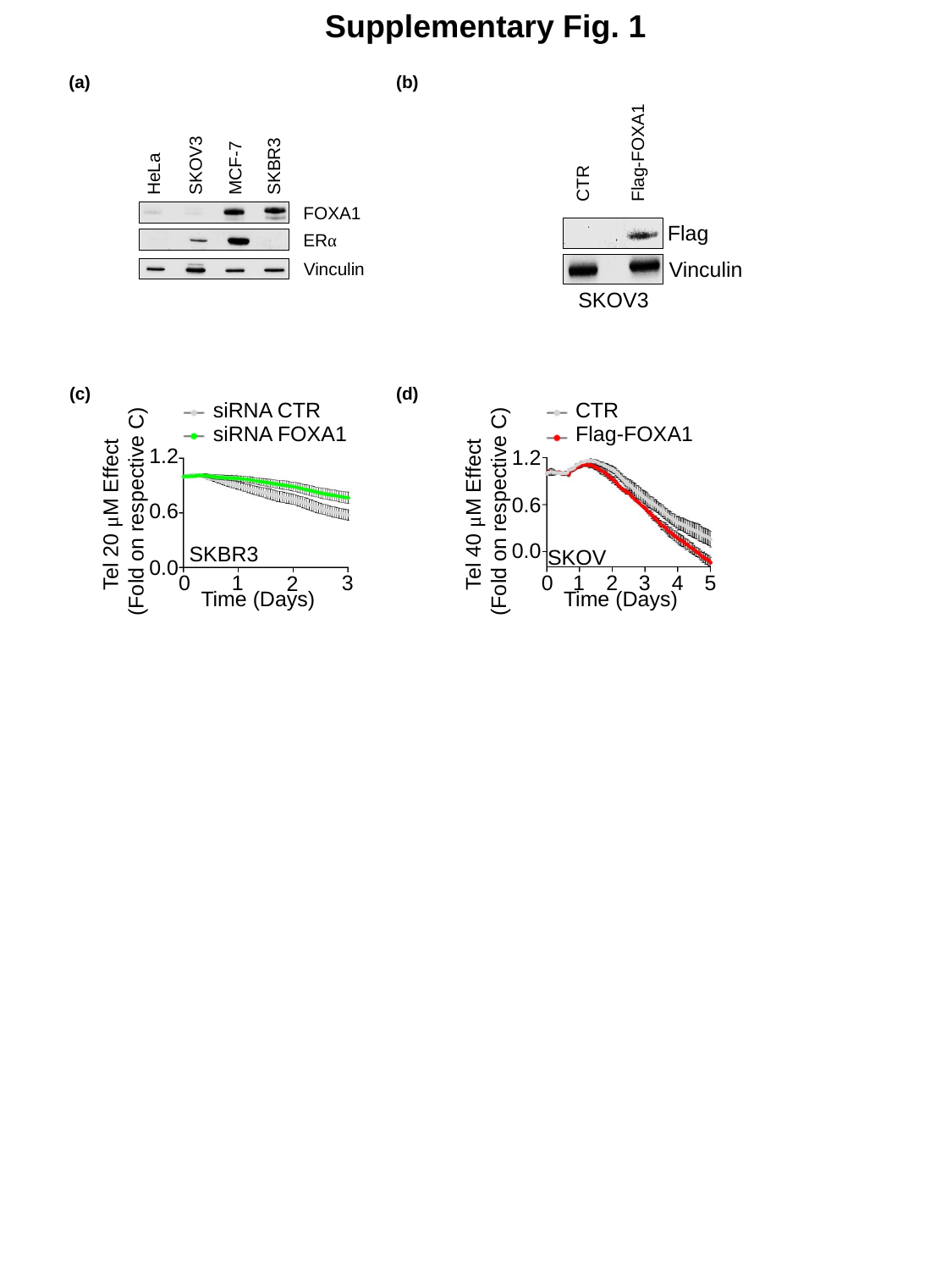

Supplementary Fig. 1
(a)
(b)
Flag-FOXA1
CTR
Flag
Vinculin
SKOV3
SKOV3
SKBR3
MCF-7
HeLa
FOXA1
ERα
Vinculin
(c)
(d)
siRNA CTR
siRNA FOXA1
1.2
Tel 20 μM Effect
(Fold on respective C)
0.6
SKBR3
0.0
0
1
3
2
 Time (Days)
CTR
Flag-FOXA1
1.2
Tel 40 μM Effect
(Fold on respective C)
0.6
0.0
SKOV
0
1
2
3
4
5
 Time (Days)

## Slide 2
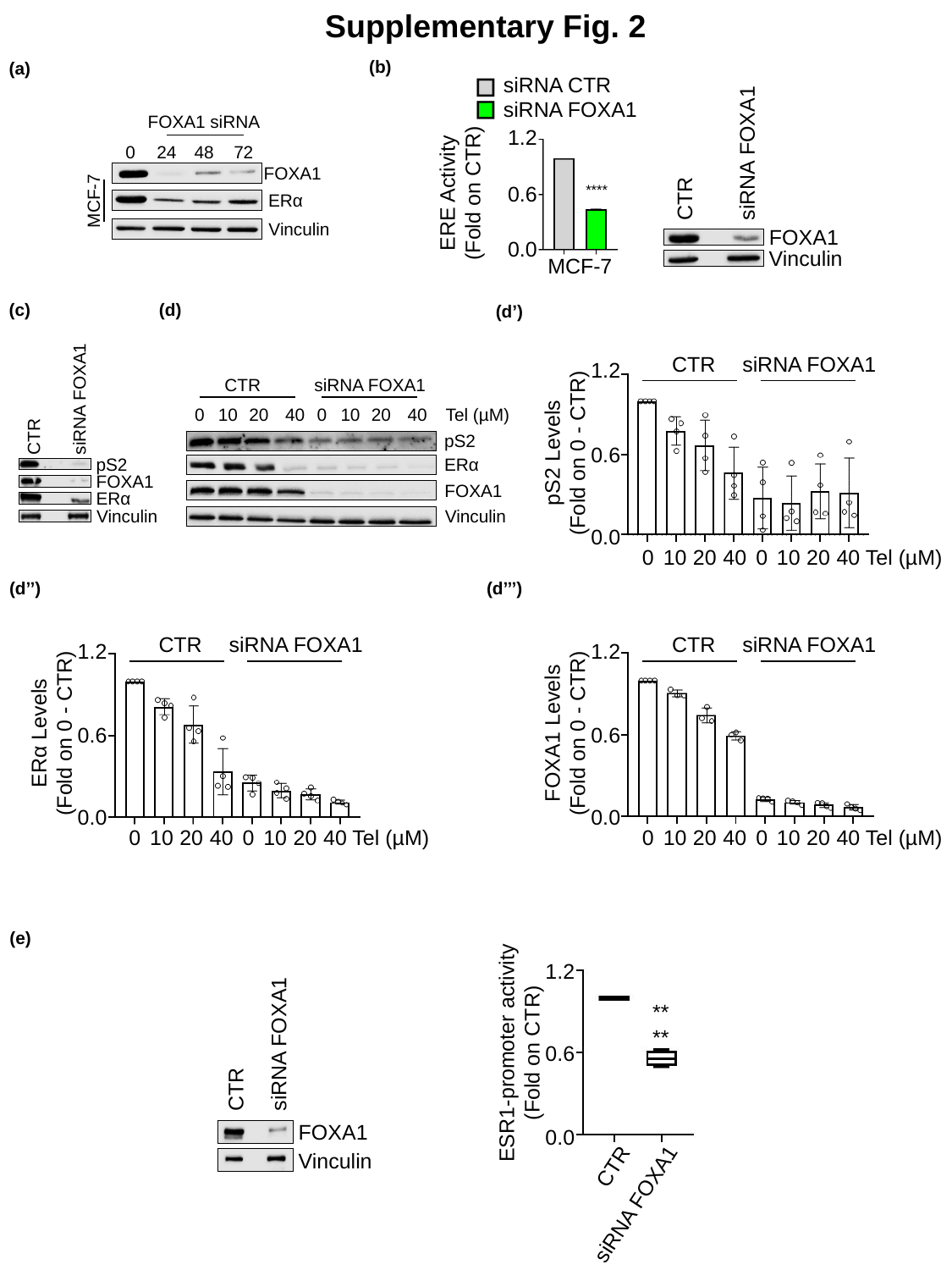

Supplementary Fig. 2
(b)
(a)
siRNA CTR
siRNA FOXA1
1.2
ERE Activity
(Fold on CTR)
****
0.6
0.0
MCF-7
siRNA FOXA1
CTR
FOXA1
Vinculin
FOXA1 siRNA
0
24
48
72
FOXA1
MCF-7
ERα
Vinculin
(c)
(d)
(d’)
siRNA FOXA1
CTR
pS2
FOXA1
ERα
Vinculin
CTR
siRNA FOXA1
1.2
pS2 Levels
(Fold on 0 - CTR)
0.6
0.0
0
10
20
40
0
10
20
40
Tel (µM)
CTR
siRNA FOXA1
0
10
20
40
0
10
20
40
Tel (µM)
pS2
ERα
FOXA1
Vinculin
(d’’)
(d’’’)
CTR
siRNA FOXA1
1.2
ERα Levels
(Fold on 0 - CTR)
0.6
0.0
0
10
20
40
0
10
20
40
Tel (µM)
CTR
siRNA FOXA1
1.2
FOXA1 Levels
(Fold on 0 - CTR)
0.6
0.0
0
10
20
40
0
10
20
40
Tel (µM)
(e)
1.2
**
**
ESR1-promoter activity
(Fold on CTR)
0.6
0.0
CTR
siRNA FOXA1
siRNA FOXA1
CTR
FOXA1
Vinculin

## Slide 3
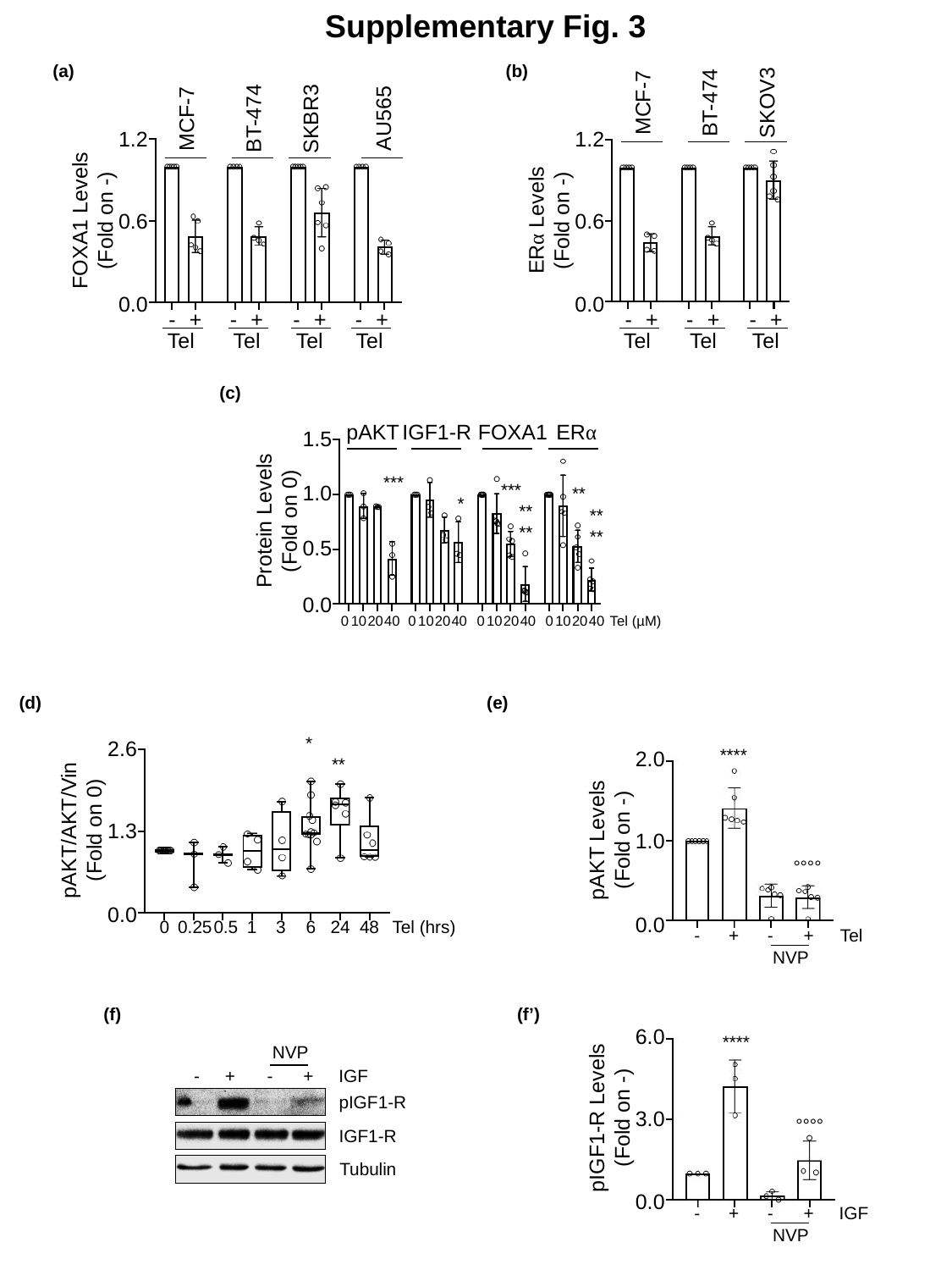

Supplementary Fig. 3
(a)
(b)
SKOV3
BT-474
MCF-7
1.2
ERα Levels
(Fold on -)
0.6
0.0
-
+
-
+
-
+
Tel
Tel
Tel
SKBR3
BT-474
MCF-7
AU565
1.2
FOXA1 Levels
(Fold on -)
0.6
0.0
-
+
-
+
-
+
-
+
Tel
Tel
Tel
Tel
(c)
pAKT
IGF1-R
FOXA1
ERα
1.5
1.0
Protein Levels
(Fold on 0)
0.5
0.0
0
10
20
40
0
10
20
40
0
10
20
40
0
10
20
40
Tel (µM)
***
***
**
*
**
**
**
**
(d)
(e)
*
2.6
**
pAKT/AKT/Vin
(Fold on 0)
1.3
0.0
0
0.25
0.5
1
3
6
24
48
Tel (hrs)
****
2.0
pAKT Levels
(Fold on -)
1.0
0.0
-
+
-
+
Tel
NVP
°°°°
(f)
(f’)
6.0
pIGF1-R Levels
(Fold on -)
3.0
0.0
-
+
-
+
IGF
NVP
****
NVP
-
+
-
+
IGF
pIGF1-R
IGF1-R
Tubulin
°°°°

## Slide 4
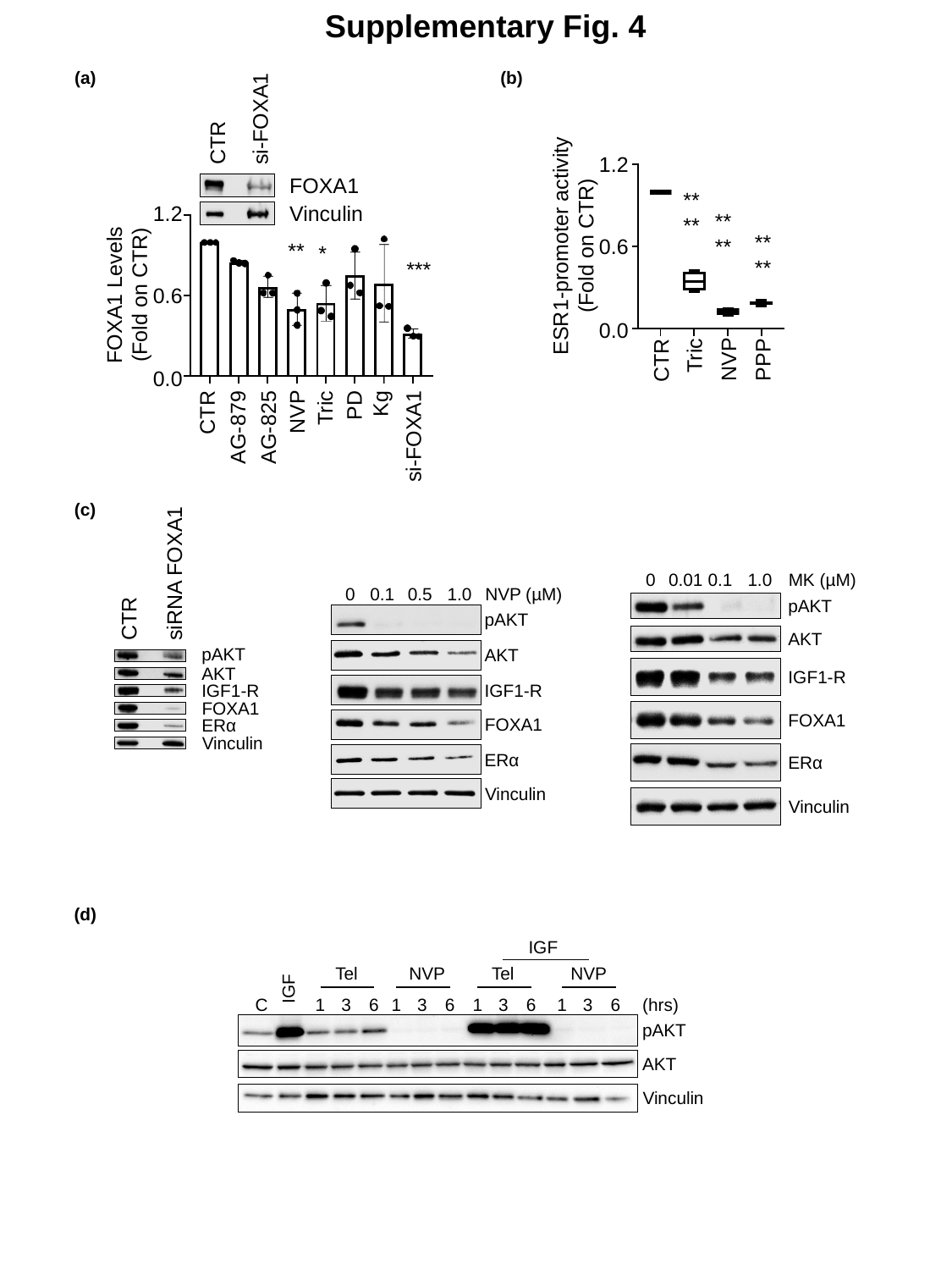

Supplementary Fig. 4
si-FOXA1
CTR
FOXA1
Vinculin
1.2
**
*
***
FOXA1 Levels
(Fold on CTR)
0.6
0.0
Kg
PD
Tric
NVP
CTR
AG-879
AG-825
si-FOXA1
(a)
(b)
1.2
**
**
**
**
ESR1-promoter activity
(Fold on CTR)
**
**
0.6
0.0
Tric
NVP
PPP
CTR
siRNA FOXA1
CTR
pAKT
AKT
IGF1-R
FOXA1
ERα
Vinculin
(c)
0
0.01
0.1
1.0
MK (µM)
pAKT
AKT
IGF1-R
FOXA1
ERα
Vinculin
0
0.1
0.5
1.0
NVP (µM)
pAKT
AKT
IGF1-R
FOXA1
ERα
Vinculin
(d)
IGF
Tel
NVP
Tel
NVP
IGF
C
1
3
6
1
3
6
1
3
6
1
3
6
(hrs)
pAKT
AKT
Vinculin

## Slide 5
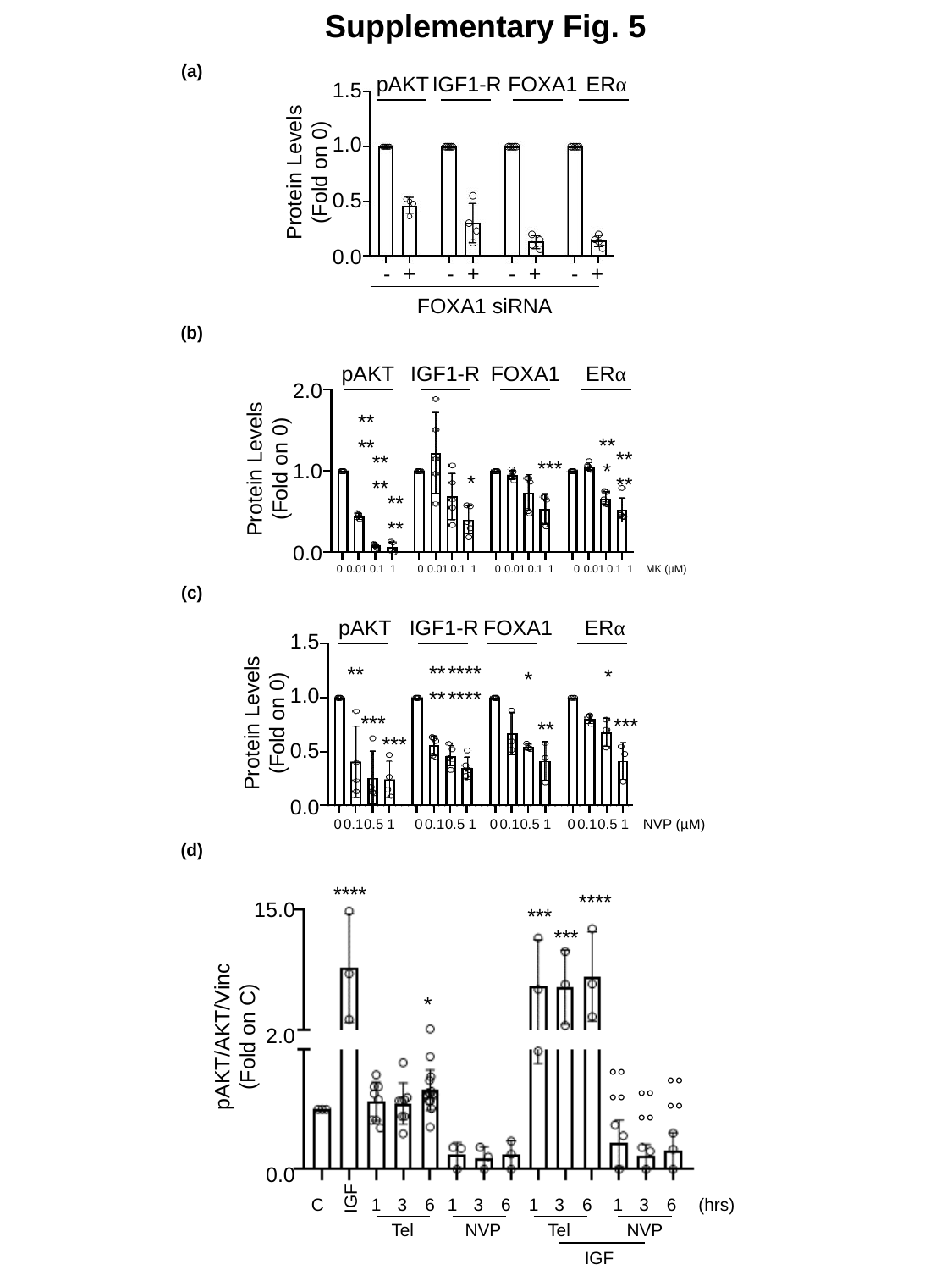

Supplementary Fig. 5
(a)
pAKT
IGF1-R
FOXA1
ERα
1.5
1.0
Protein Levels
(Fold on 0)
0.5
0.0
-
+
-
+
-
+
-
+
FOXA1 siRNA
(b)
pAKT
IGF1-R
FOXA1
ERα
2.0
**
**
**
*
Protein Levels
(Fold on 0)
**
**
**
**
***
1.0
*
**
**
0.0
0
0.01
0.1
1
0
0.01
0.1
1
0
0.01
0.1
1
0
0.01
0.1
1
MK (µM)
(c)
pAKT
IGF1-R
FOXA1
ERα
1.5
1.0
Protein Levels
(Fold on 0)
0.5
0.0
0
0.1
0.5
1
0
0.1
0.5
1
0
0.1
0.5
1
0
0.1
0.5
1
NVP (µM)
**
**
**
**
**
**
**
*
*
***
***
**
***
(d)
****
****
15.0
pAKT/AKT/Vinc
(Fold on C)
2.0
0.0
IGF
C
1
3
6
1
3
6
1
3
6
1
3
6
(hrs)
Tel
NVP
Tel
NVP
IGF
***
***
*
°°
°°
°°
°°
°°
°°

## Slide 6
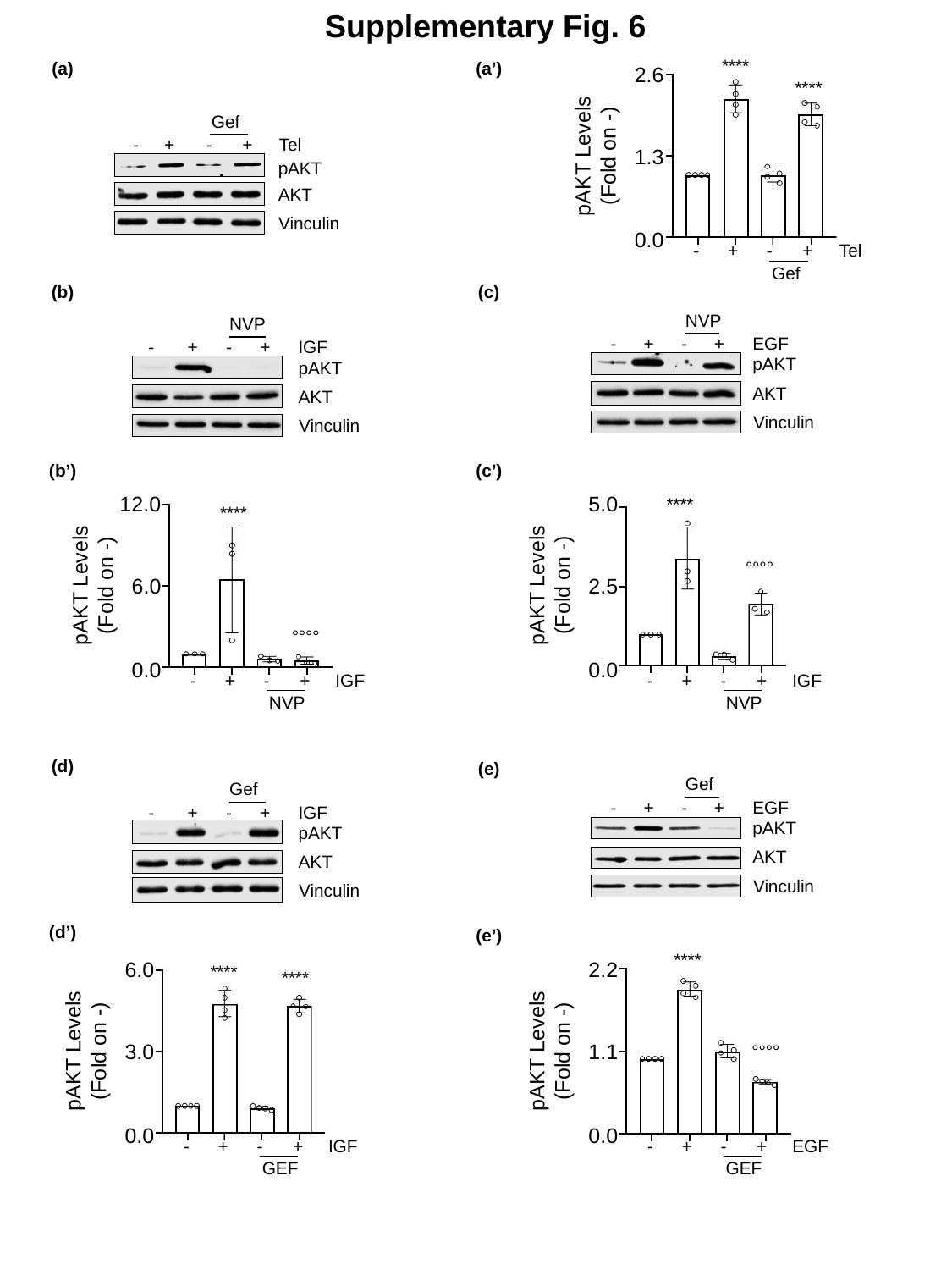

Supplementary Fig. 6
****
(a)
(a’)
2.6
pAKT Levels
(Fold on -)
1.3
0.0
-
+
-
+
Tel
Gef
****
Gef
-
+
-
+
Tel
pAKT
AKT
Vinculin
(b)
(c)
NVP
-
+
-
+
EGF
pAKT
AKT
Vinculin
NVP
-
+
-
+
IGF
pAKT
AKT
Vinculin
(b’)
(c’)
12.0
pAKT Levels
(Fold on -)
6.0
0.0
-
+
-
+
IGF
NVP
5.0
pAKT Levels
(Fold on -)
2.5
0.0
-
+
-
+
IGF
NVP
****
****
°°°°
°°°°
(d)
(e)
Gef
-
+
-
+
EGF
pAKT
AKT
Vinculin
Gef
-
+
-
+
IGF
pAKT
AKT
Vinculin
(d’)
(e’)
****
6.0
pAKT Levels
(Fold on -)
3.0
0.0
-
+
-
+
IGF
GEF
2.2
pAKT Levels
(Fold on -)
1.1
0.0
-
+
-
+
EGF
GEF
****
****
°°°°

## Slide 7
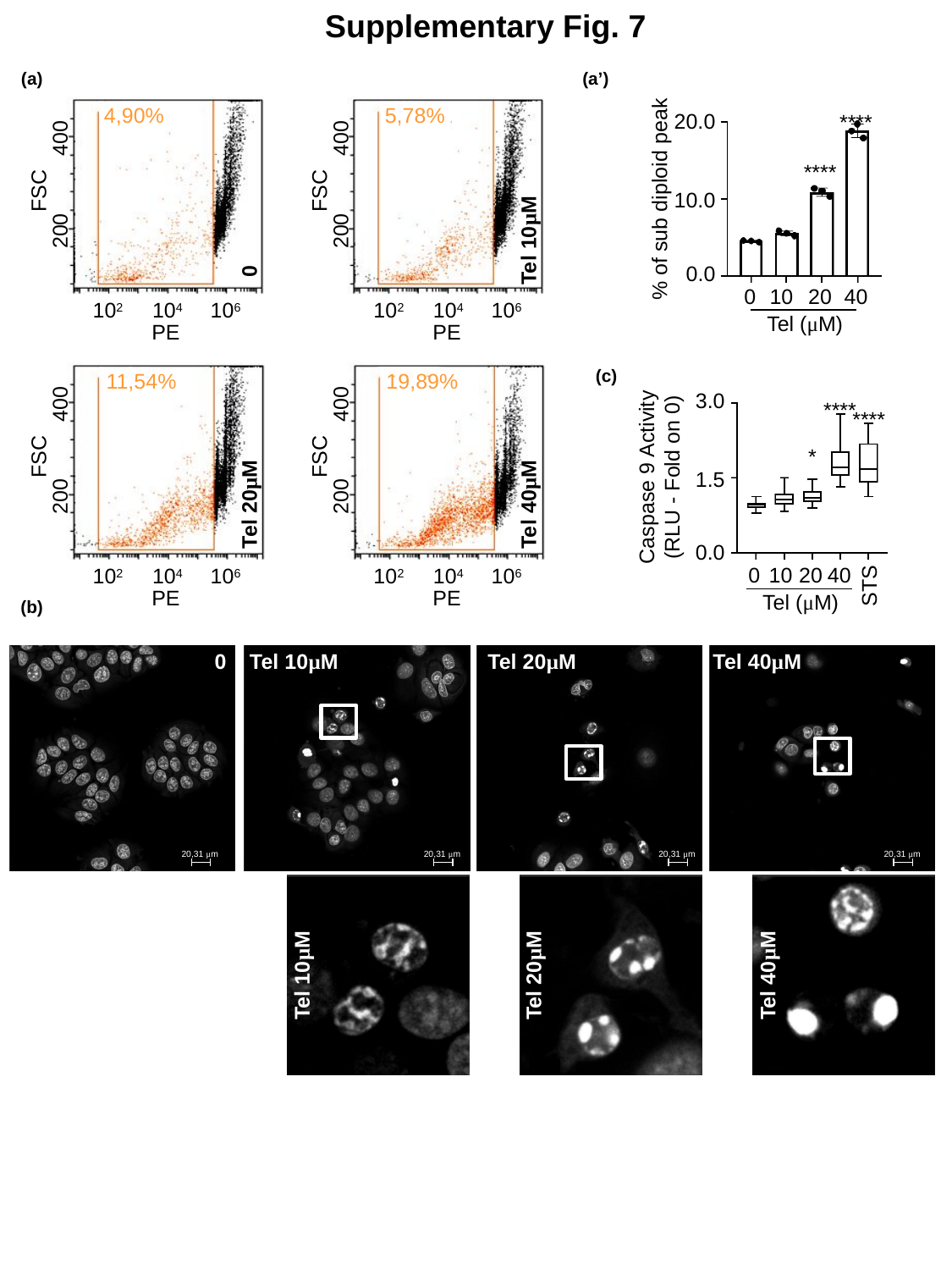

Supplementary Fig. 7
(a)
(a’)
20.0
****
****
% of sub diploid peak
10.0
0.0
0
10
20
40
 Tel (μM)
4,90%
400
FSC
200
102
104
106
PE
0
5,78%
400
FSC
200
102
104
106
PE
Tel 10μM
(c)
11,54%
400
FSC
200
102
104
106
PE
Tel 20μM
19,89%
400
FSC
200
102
104
106
PE
Tel 40μM
3.0
****
****
*
Caspase 9 Activity
(RLU - Fold on 0)
1.5
0.0
0
10
20
40
STS
 Tel (μM)
(b)
0
Tel 10μM
Tel 20μM
Tel 40μM
20,31 μm
20,31 μm
20,31 μm
20,31 μm
Tel 10μM
Tel 20μM
Tel 40μM

## Slide 8
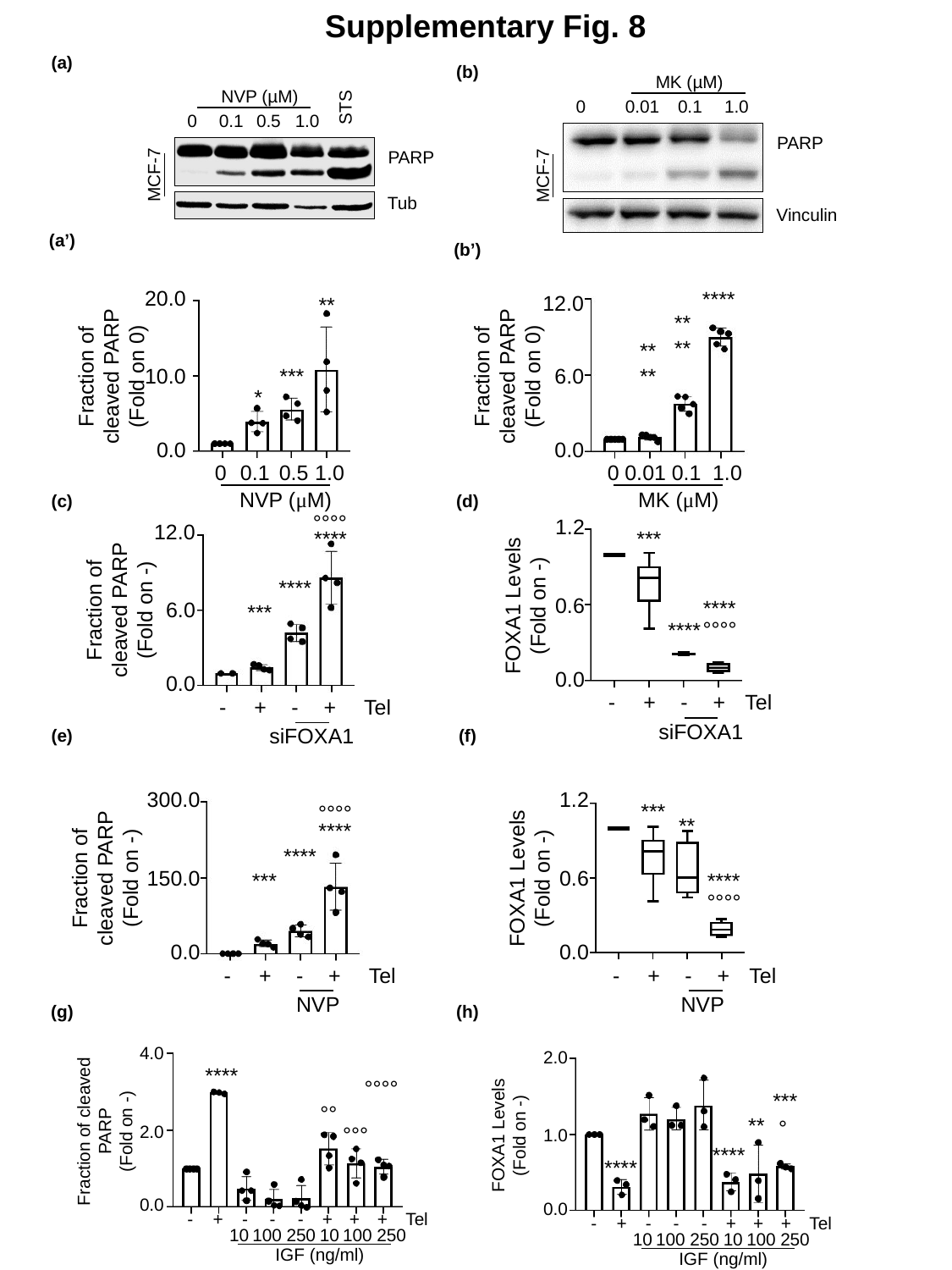

Supplementary Fig. 8
(a)
(b)
MK (µM)
0
0.01
0.1
1.0
PARP
MCF-7
Vinculin
NVP (µM)
STS
0
0.1
0.5
1.0
PARP
MCF-7
Tub
(a’)
(b’)
20.0
**
Fraction of cleaved PARP
(Fold on 0)
***
10.0
*
0.0
0
0.1
0.5
1.0
 NVP (μM)
****
12.0
**
**
**
**
Fraction of cleaved PARP
(Fold on 0)
6.0
0.0
0
0.01
0.1
1.0
 MK (μM)
(c)
(d)
°°°°
12.0
****
Fraction of cleaved PARP
(Fold on -)
****
6.0
***
0.0
-
+
-
+
Tel
siFOXA1
1.2
***
FOXA1 Levels
(Fold on -)
0.6
****
°°°°
****
0.0
-
+
-
+
Tel
siFOXA1
(e)
(f)
300.0
****
Fraction of cleaved PARP
(Fold on -)
****
150.0
***
0.0
-
+
-
+
Tel
NVP
°°°°
1.2
***
**
FOXA1 Levels
(Fold on -)
0.6
****
°°°°
0.0
-
+
-
+
Tel
NVP
(g)
(h)
4.0
****
°°°°
Fraction of cleaved PARP
(Fold on -)
°°
2.0
°°°
0.0
-
+
-
-
-
+
+
+
Tel
10
100
250
10
100
250
IGF (ng/ml)
2.0
***
**
FOXA1 Levels
(Fold on -)
°
1.0
****
****
0.0
-
+
-
-
-
+
+
+
Tel
10
100
250
10
100
250
IGF (ng/ml)

## Slide 9
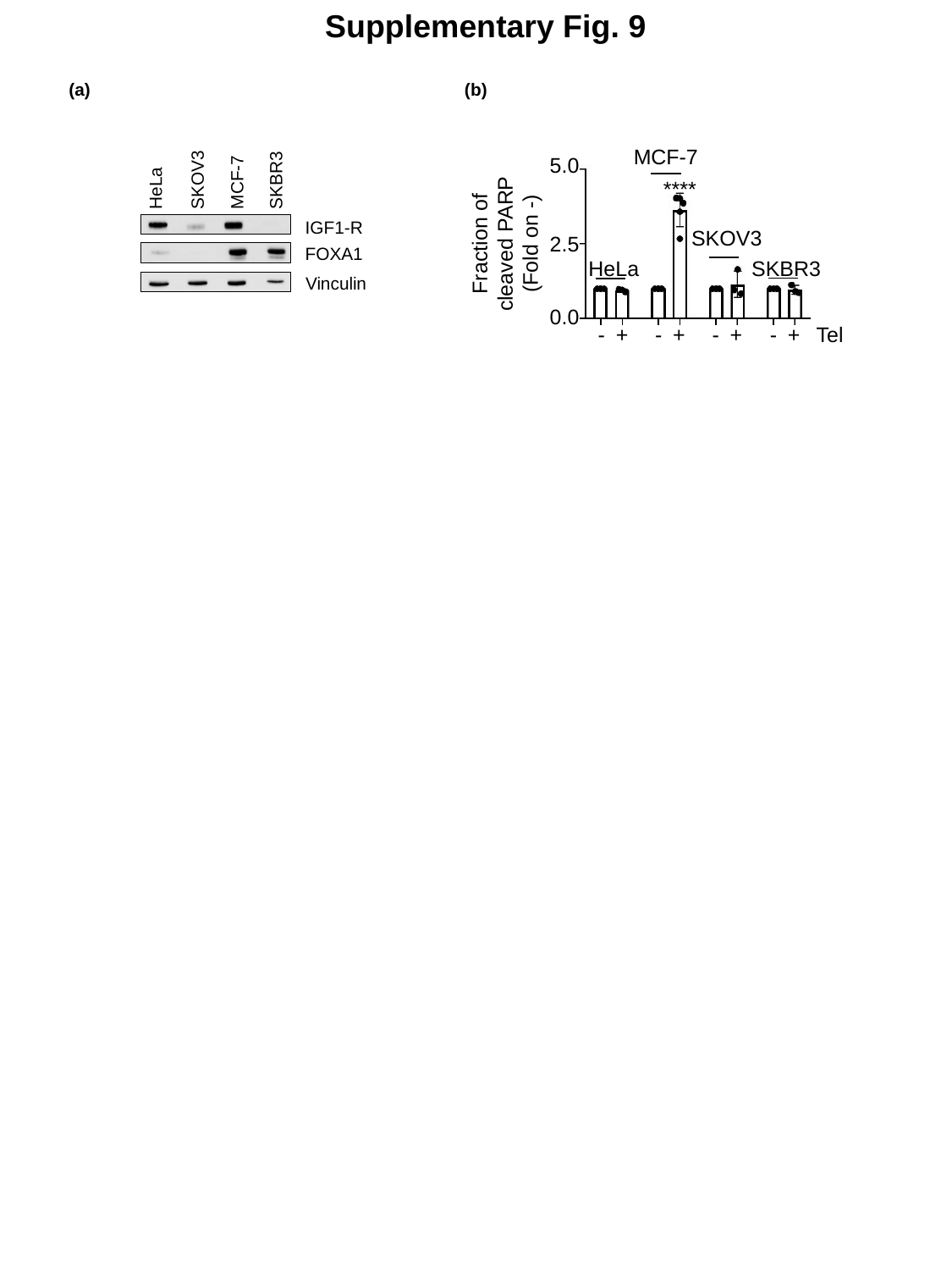

Supplementary Fig. 9
(a)
(b)
MCF-7
5.0
****
Fraction of cleaved PARP
(Fold on -)
SKOV3
2.5
HeLa
SKBR3
0.0
-
+
-
+
-
+
-
+
Tel
SKOV3
SKBR3
MCF-7
HeLa
IGF1-R
FOXA1
Vinculin
